# Impact of a multifaceted intervention to improve antibiotic prescribing: a pragmatic cluster-randomised controlled trial

**DOI:** 10.1186/s13756-020-00857-9

**Published:** 2020-12-07

**Authors:** Adolfo Figueiras, Paula López-Vázquez, Cristian Gonzalez-Gonzalez, Juan Manuel Vázquez-Lago, María Piñeiro-Lamas, Ana López-Durán, Coro Sánchez, María Teresa Herdeiro, Maruxa Zapata-Cachafeiro, on behalf of the GREPHEPI Group, Francisco Caamaño, Juan J. Gestal-Otero, Margarita Taracido, Elena Lopez-Gonzalez, Isabel Sastre, Ana Estany, Angel Salgado

**Affiliations:** 1 Department of Preventive Medicine and Public Health, University of Santiago de Compostela, 15786 Santiago de Compostela, Spain; 2 Consortium for Biomedical Research in Epidemiology and Public Health (CIBER en Epidemiología y Salud Pública- CIBERESP), Santiago de Compostela, Spain; 3 Health Research Institute of Santiago de Compostela (IDIS), Santiago de Compostela, Spain; 4 Department of Clinical Psychology and Psychobiology, University of Santiago de Compostela, Santiago de Compostela, Spain; 5 Pontevedra Primary Care Service, SERGAS Eoxi Pontevedra-Salnés, Pontevedra, Spain; 6 Department of Medical Sciences & Institute for Biomedicine – iBiMED, University of Aveiro, Aveiro, Portugal

**Keywords:** Primary care, Physicians, Attitudes, Microbial resistances, Antibiotics, Inappropriate prescribing, Educational intervention

## Abstract

**Objectives:**

This study sought to assess the effectiveness and return on investment (ROI) of a multifaceted intervention aimed at improving antibiotic prescribing for acute respiratory infections in primary care.

**Design:**

Large-sized, two-arm, open-label, pragmatic, cluster-randomised controlled trial.

**Setting:**

All primary care physicians working for the Spanish National Health Service (NHS) in Galicia (region in north-west Spain).

**Participants:**

The seven spatial clusters were distributed by unequal randomisation (3:4) of the intervention and control groups. A total of 1217 physicians (1.30 million patients) were recruited from intervention clusters and 1393 physicians (1.46 million patients) from control clusters.

**Interventions:**

One-hour educational outreach visits tailored to training needs identified in a previous study; an online course integrated in practice accreditation; and a clinical decision support system.

**Main outcome measures:**

Changes in the ESAC (European Surveillance of Antimicrobial Consumption) quality indicators for outpatient antibiotic use. We used generalised linear mixed and conducted a ROI analysis to ascertain the overall cost savings.

**Results:**

Median follow-up was 19 months. The adjusted effect on overall antibiotic prescribing attributable to the intervention was − 4.2% (95% CI: − 5.3% to − 3.2%), with this being more pronounced for penicillins − 6.5 (95% CI: − 7.9% to − 5.2%) and for the ratio of consumption of broad- to narrow-spectrum penicillins, cephalosporins, and macrolides − 9.0% (95% CI: − 14.0 to − 4.1%). The cost of the intervention was €87 per physician. Direct savings per physician attributable to the reduction in antibiotic prescriptions was €311 for the NHS and €573 for patient contributions, with an ROI of €2.57 and €5.59 respectively.

**Conclusions:**

Interventions designed on the basis of gaps in physicians’ knowledge of and attitudes to misprescription can improve antibiotic prescribing and yield important direct cost savings.

*Trial registration*: Current Controlled Trials ISRCTN24158380. Registered 5 February 2009.

## Background

Antibiotic-resistant pathogens have emerged and spread worldwide, to the point that they pose a major public health threat [1, 2]. This loss of efficacy against common pathogens has increased morbidity, mortality and health care costs [3, 4] in low- and high-income countries alike. Although there is no doubt about the link between excessive consumption of antibiotics and antimicrobial resistance, at a global level antibiotic use nonetheless continues to rise [2].

Most antibiotic prescriptions are issued to outpatients: [5] in 2011, non-hospital antibiotic use in the Spanish National Health Service (NHS) in Galicia totalled 20.9 defined daily doses (DDD) per 1000 inhabitants per day [6]. In addition, there are prescriptions issued by private physicians and drugs dispensed without a medical prescription, [7] which would go to increase mean consumption of antibiotics in Spain to almost 30 DDD, one of the highest rates in Europe [6]. At least one third of all prescriptions are for treatment of acute respiratory infections (ARIs) [8, 9], but only half of these are thought to be appropriate [9]. While the reasons for this divergence between evidence-based guidelines and general practitioners’ prescribing behaviour are not clear, [10] they are crucial when it comes to designing strategies to help improve antibiotic prescribing [11].

In order to identify the reasons behind the inappropriate prescribing of antibiotics for ARIs in the north-western region of Spain (2.7 million inhabitants) and use these to develop a purpose-designed intervention to tackle the problem, this project was undertaken in stages. Stage one involved carrying out a qualitative study [12] to ascertain physicians’ knowledge and attitudes regarding the use of antibiotics, and, on this basis, develop the first fully-validated questionnaire to evaluate such knowledge and attitudes [13]. Secondly, using this questionnaire, an observational study was conducted [14] to identify *gaps in knowledge and attitudes* associated with inappropriate antibiotic prescriptions by primary care physicians. Thirdly, based on these results, a multifaceted intervention (outreach visit, online course, patient support materials, internet-based clinical decision support system) was implemented to improve antibiotic prescribing for ARIs [15]. The final stage consisted of performing a large-sized cluster-randomised trial to assess the intervention.

Many trials have assessed the effectiveness of educational interventions to improve antibiotic use in primary care, mostly with moderate effects, [16] thereby conveying the feeling that these interventions have not been altogether successful [17]. In an environment in which there are seemingly no definitive solutions for radically reducing resistance, these interventions could have great relevance for policy makers when it comes to improving prescribing and yielding cost savings over a relatively short time horizon [18]. Even so, few well-designed trials have assessed the economic aspects of interventions to improve antibiotic prescribing [19]. Accordingly, this study sought to show the effectiveness (improved prescriptions) and efficacy (direct cost savings) of a multifaceted intervention aimed at improving antibiotic prescribing for ARIs in primary care [20].

## Methods

### Settings

Galicia is a region situated in the north-west of Spain. It has 2.7 million inhabitants, a quarter of whom are over 65 years of age; 98% of the population is covered by the the Spanish National Health Service (NHS), which is almost fully funded by taxes and comes predominantly within the public sector. Provision of all health services, other than pharmaceutical, is free of charge at the point of delivery.

### Study population

The study population comprised all primary care physicians working at primary care health centres operated by the Spanish NHS in Galicia at the date of study (N ≅ 3673).The following were excluded: (1) temporary staff and medical residents in training, since such persons register very low prescribing levels for short periods, and including them might generate numerical instability in the indicators assessed; and, (2) physicians exclusively assigned to emergencies, since they do not have a designated number of listed patients, thus rendering it impossible to calculate indicators that require the number of patients attended as their denominator.

### Study design

**Fig. 1:**
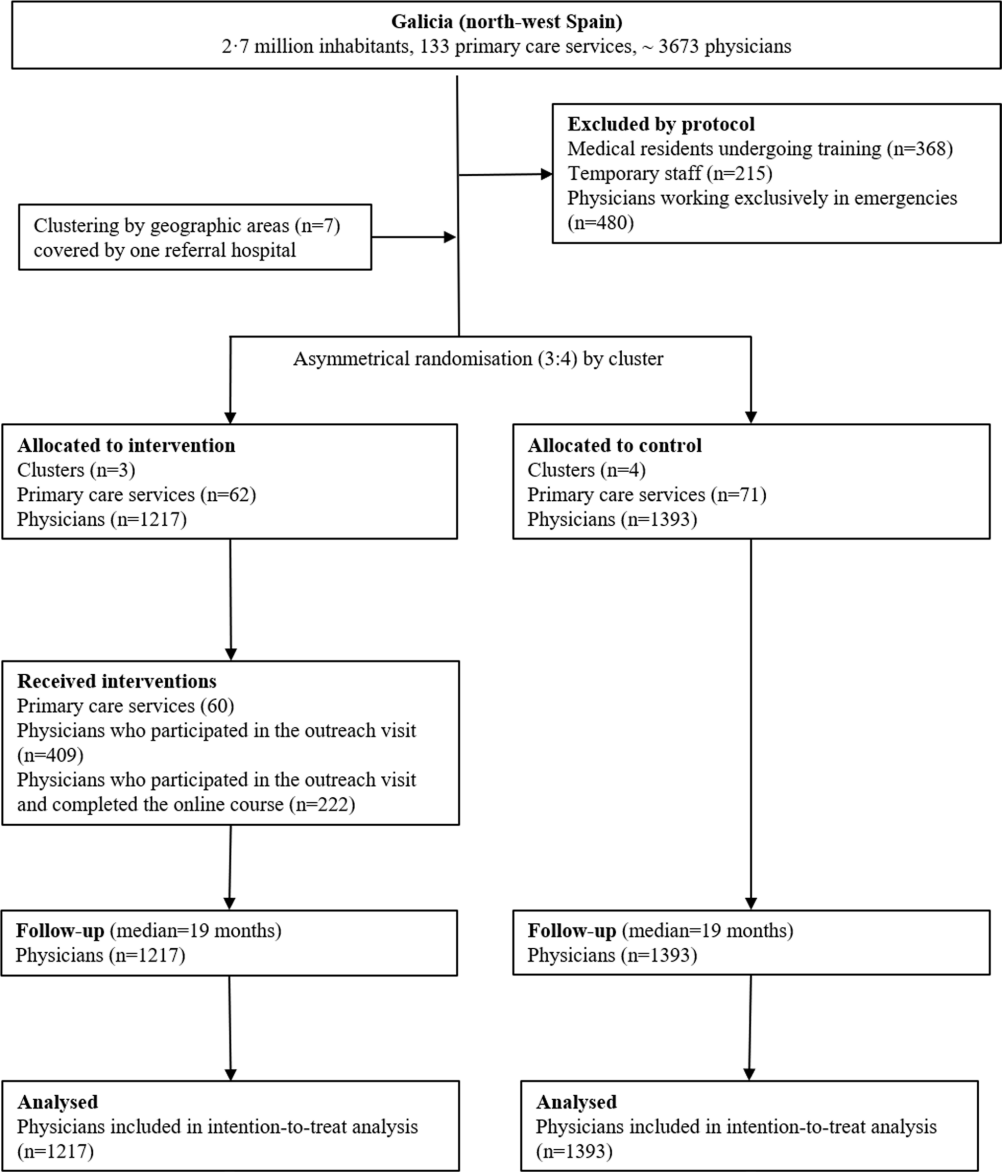
Flow of participants through the study

The study is reported according to the CONSORT statement for cluster randomised controlled trials. We conducted a *large sized, pragmatic, two-arm, prospective, cluster-randomised controlled trial.* To minimise the presence of cross-contamination between the intervention and control groups, the study area (Galicia) was divided into seven spatial clusters in accordance with the distribution of health-service management areas. Each cluster contained all the physicians who worked at outpatient centres in the selected geographic area. For economic efficiency, the clusters were distributed by unequal randomization with an intervention:control group ratio of 3:4 (see Fig. 1). This clusters were assigned with a computer-generated procedure.

### Intervention

We designed a multifaceted low-cost intervention, feasible academically and consisting of an outreach visit, an online course, an internet-based clinical decision support system, and an information leaflet for patients. The intervention was designed on the basis of gaps of the study population in knowledge and inappropriate attitudes associated with inappropriate prescription detected by previous studies [12, 14].

Firstly, to tackle *the perception that antibiotic resistance is not a problem at a community level,* the outreach visit highlighted: (i) data on the impact of resistance (mortality, morbidity); (ii) data on antibiotic use in Spain and Galicia (three times higher than The Netherlands); and (iii) the relationship between antibiotic use and resistance, as shown by previous studies.

Secondly, to address *complacency* (attitude that motivates the prescribing of antibiotics to fulfil professionals’ perceptions of their patients’ expectations), the outreach visit stressed: (i) the importance of communication skills (assertiveness, empathy, negotiation) [21]; and, (ii) the effectiveness of delayed prescribing (prescription with the physician advising the patient to collect it in a few days’ time, if the symptoms show no improvement) [22]. In addition, patient-support materials were distributed in the waiting rooms (see Additional file 1, in Spanish).

Thirdly, to address *fear* (attitude relating to fear of possible future complications in the patient) and *insufficient knowledge*, we encouraged delayed prescribing, held a training course (online integrated practice-accreditation course, see Additional file 2, in Spanish), and offered access to an internet-based clinical decision support system.

The interventions were implemented from December 2011 to July 2012. The follow-up period for each physician began once the outreach visit had been conducted at his/her health centre. Follow-up continued until September 2013. The clusters of the control group were matched by proximity with that of the intervention group, and the follow-up was started with the first intervention of the matched intervention group. As our data source, we used monthly administrative prescription-dispensing data supplied by the Spanish NHS in Galicia (indications or diagnoses for which antibiotics are prescribed was unavailable). This database carries no record of the indications or diagnoses for which antibiotics are prescribed: it only records prescriptions that have been dispensed at a pharmacy, with the result that undispensed prescriptions (e.g., due to delayed prescribing) are not included.

A fuller description of each of the activities included in the multifaceted intervention (which was standard for all participants) is now given below and can also be consulted a timeline of each them in Additional file 3.

- The outreach visit consisted of a 1-h presentation made during weekly staff meetings, so as to ensure that the greatest number of physicians would be present [23]. The groups consisted of 3 to 18 physicians, and almost all visits took place between December 2011 and July 2012. The presentation was given by a team pharmacist (CGG) who belonged to the University of Santiago research team and was not known to the physicians. The visit included an offer to participate in an online integrated practice-accreditation course (see Additional file 4).
- The outreach visit was combined with a ten-hour online integrated practice-accreditation course (blended learning), designed to update knowledge of respiratory infection management on the basis of the Spanish Family & Community Medicine Society Guidelines [24]. The course not only enjoyed the Family & Community Medicine Society’s support, but was also accredited by the continuing medical education system and thus counted towards integrated in-practice accreditation. Three editions of the course were held from January to September 2012. The online course’s appearance and an example of how it operates can be seen in the Additional file 2 (in Spanish).
- All on-line course participants had right of access, during and up to 2 years after the course, to an internet-based clinical decision support system for respiratory infection management, based on the Spanish Family & Community Medicine Society’s algorithms [24].
- Furthermore, patient-support materials for self-care of respiratory infections, drawn up on the basis of those designed by the European Centre for Disease Control (ECDC), were placed by the visiting team member in the waiting rooms of all health centres assigned to the intervention group. The patient-support materials can be seen in the supplementary document.
- Seven months after conclusion of the outreach visits, an e-mail was circulated to the physicians at the health centres assigned to the intervention clusters, reminding them of the content of the training session and the possibility of using the internet-based clinical decision support system.

Physicians belonging to the control group did not participate in any part of the intervention and, only intervention-group physicians who attended the outreach visit enjoyed access to the course and clinical decision support system, and received the reminder e-mail.

### Outcome measure and follow-up

To assess prescribing quality, we used the quality indicators for outpatient antibiotic use developed by the European Surveillance of Antimicrobial Consumption (ESAC) project [25]. These indicators are reproducible, validated and external (proposed by other researchers), and we chose them to avoid any arbitrariness or opportunistic choice of output.

The ESAC quality indicators for outpatient antibiotic use assess: (1) the total volume of antibiotic use (DID of antibiotics for systemic use); (2) antibiotic use by group (cephalosporins macrolides, lincosamides and streptogramins, quinolones); and, (3) the percentage of prescriptions of second-line antibiotics (e.g., 3rd- and 4th-generation of broad-spectrum cephalosporins) over total or over first-line antibiotics. In the absence of clinical data, high percentages of these indicators could indicate inappropriate prescription, due the limited evidence of additional clinical benefit of second-line over first-line antibiotics for the most common indications in primary care [25].

Moreover, the use of these indicators, which were calculated per physician per month, lent our study a number of strengths: (1) their use ruled out any possible opportunistic selection of indicators; (2) they facilitated comparability with similar studies [26]; and (3) the non-inclusion of drug indication prevented possible diagnostic shift [27].

The costs of prescribing were also obtained from Spanish NHS in Galicia.

### Ethics statement

The study was approved by the Galician Ethics Committee (code number 2007/107). During the briefing session, all intervention-group physicians were informed of the purpose of the study and their consent was obtained prior to participating in the different parts of the intervention. Permission to conduct the outreach visits was likewise obtained from the management of the respective health centres. The antibiotic prescription indicators were furnished by the Spanish NHS in Galicia in an anonymised format.

As no intervention was conducted on the physicians in the control group (or on those in the intervention group who failed to attend the sessions) and their data were supplied in an anonymised format, these individuals were deemed not to be research participants from an ethical standpoint [28] and their informed consent was not considered necessary.

The trial was registered under ISRCTN registry number ISRCTN24158380.

### Statistical analysis

All statistical analyses were performed on an *intention-to-treat* basis. Linear mixed models (LMMs) fitted by restricted maximum likelihood, were applied to the statistical analysis. All analyses were performed using the lme4 package for the free R Statistical Software (version 3.1.1). The degrees of freedom used to calculate the confidence intervals and the *p*-values were corrected by Satterthwaite’s method (lmerTest package) in order to minimise their influence due to the small number of clusters.

The models were adjusted for the covariates of Table 1 and trend/seasonality (due to the fact that antibiotic prescribing may have a baseline trend, and, in addition, has a seasonal component). Hence, a trend/seasonality variable was created, such that it took a value of 0 for the warm months (April to September) of the first year, a value of 1 for the cold months (October to March) of that same year, a value of 2 for the cold months of the following year, and so on successively.

**Table 1:** Baseline characteristics of randomised primary care services^a^ and physicians, by intervention group

	Intervention (3 clusters, ***n*** = 1217 physicians)	Control (4 clusters, ***n*** = 1393 physicians)
**Primary care services, n**	62	71
**Primary care services: habitat, n (%)**		
Urban	22 (35.5)	20 (28.2)
Rural	40 (64.5)	51 (71.8)
**Primary care services: habitat, n (%)**		
Coast	33 (53.2)	32 (45.1)
Interior	29 (46.8)	39 (54.9)
**Primary care services: residency training, n (%)**	23 (37.1)	18 (25.4)
**Physicians, by primary care centre, n (%)**		
Median (P25 – P75)	19 (10–27.5)	16 (10–26)
≤ 10 physicians	17 (27.4)	19 (26.8)
10–20 physicians	16 (25.8)	22 (31)
> 20 physicians	29 (46.8)	30 (42.3)
**Size of patient list, by group (sum)**	1,515,330	1,785,960
**Size of patient list, by physician**		
Median (IQR)	1309 (1018–1460)	1357 (1070–1501)
Mean (SD)	1245 (321.2)	1282 (303.3)
**Size of list of patients aged > 65 years, by physician**		
Median (IQR)	399 (296–490)	441 (321–536)
**Months: number at baseline worked by physician**		
Median (IQR)	49 (41–51)	48 (38–49)
**Antibiotics for systemic use at baseline**		
Number (in millions) of prescription-dispensing (sum)	1.33	1.42
DIDs per year (IQR)	15.9 (15.8–16.0)	14.9 (14.8–15.0)

*Abbreviations*. *IQR* Interquartile range, *SD* Standard deviation, *DID* Defined daily doses per 1000 inhabitants

^a^ A service can be formed by a health center (median number of health centers per service (IQR): 3 (1–5))

Moreover, a power analysis of the total DID prescribed model was performed using the R package simr.

### Interpretation

The estimates of interest were calculated by means of the intervention (intervention vs control) × period (pre- vs post-intervention) interaction terms, which represented absolute changes (i.e. between pre- and post-intervention) in prescribing indicators during the post-intervention period, with respect to concurrent controls. This makes it possible to obtain effects adjusted for the potential differences between the groups and for the pre-intervention values of the outcome [23].

### Economic analysis

LMMs were also used for cost analysis [29]. Using these, we calculated the reduction in mean costs associated with each study unit (physician) attributable to the intervention [19].

Calculations of savings were made in respect of the total cost of antibiotics (sum of NHS and patient contributions) and in respect of the NHS alone.

Lastly, we performed an economic evaluation of the intervention by reference to the ROI ratio, [30] which evaluates the financial return of an intervention against the total costs of its delivery. The ROI is the benefit minus the cost, expressed as a proportion of the cost, [18] namely:

$$ROI=\frac{Net\ benefit}{Cost}=\frac{Benefit- cost}{Cost}$$

The ROI indicates the savings (in euro) for every €1.00 spent on the intervention.

## Results

### Sample characteristics

Of a total of 3673 primary care physicians in north-west Spain, 1063 were excluded (Fig. 1). It will be seen that 631/1217 (33.6%) of potential participants attended the in-person sessions, and 222/1217 (18.2%) completed the online course. The baseline characteristics of the intervention and control groups are shown in Table 1.

### Antibiotic prescription

Table 2 shows the values of the indicators during the baseline and post-intervention periods for both groups. To control for the baseline differences, statistical models were adjusted for the baseline values of all indicators. As will be seen, in most of the indicators there is an improvement between the pre-and post-intervention period in the control group, though this is less pronounced in the control group than in the intervention group. The effect of the intervention is also controlled for by the improvement in the control group.

**Table 2:** Values for the quality indicators at baseline and across the post-intervention period for both groups

ESAC Quality Indicators [25]	Group	Period	
		Baseline (median 48 months^**b**^)	Post-intervention (median 19 months^**b**^)
1. DID of antibiotics for systemic use	Intervention	15.9 (15.8–16.0)	13.8 (13.7–14.0)
	Control	14.9 (14.8–15.0)	13.5 (13.4–13.6)
2. DID of penicillins	Intervention	9.1 (9.1–9.2)	7.9 (7.8–8.0)
	Control	8.0 (7.9–8.0)	7.3 (7.3–7.4)
3. DID of cephalosporins	Intervention	1.9 (1.9–1.9)	1.5 (1.5–1.5)
	Control	1.8 (1.8–1.8)	1.5 (1.5–1.5)
4. DID of macrolides, lincosamides and streptogramins	Intervention	1.6 (1.6–1.6)	1.4 (1.4–1.4)
	Control	1.7 (1.7–1.8)	1.6 (1.5–1.6)
5. DID of quinolones	Intervention	2.1 (2.1–2.1)	1.9 (1.8–1.9)
	Control	2.1 (2.1–2.1)	1.9 (1.9–2.0)
6. PCT of beta-lactamase sensitive penicillins^a^	Intervention	0.6 (0.6–0.7)	0.6 (0.5–0.6)
	Control	0.4 (0.4–0.4)	0.4 (0.3–0.4)
7. PCT of combinations of penicillins with beta-lactamase inhibitors ^a^	Intervention	42.1 (42.0–42.3)	40.0 (39.8–40.3)
	Control	39.0 (38.9–39.2)	38.2 (38.0–38.4)
8. PCT of 3rd- and 4th-generation cephalosporins ^a^	Intervention	4.0 (3.9–4.0)	2.8 (2.7–2.9)
	Control	4.2 (4.8–4.3)	3.3 (3.2–3.4)
9. PCT of quinolones	Intervention	13.3 (13.2–13.4)	13.4 (13.2–13.5)
	Control	14.3 (14.2–14.4)	14.3 (14.1–14.4)
10. Ratio of consumption of broad- to narrow-spectrum penicillins, cephalosporins and macrolides	Intervention	68.9 (67.6–70.1)	49.3 (47.5–51.0)
	Control	59.9 (58.8–60.9)	45.0 (43.6–46.5)

*Abbreviations*. *PCT* Percentage, *DID* Defined daily doses per 1000 inhabitants per day, *ESAC* European Surveillance of Antimicrobial Consumption

^a^ Percentage of total consumption of antibiotics for systemic use in DID

^b^ The interventions were implemented from December 2011 to July 2012

### Effect of the intervention

Table 3 shows the effect of the intervention on each of the indicators considered, in absolute values (principally DDD per 1000 inhabitants per day) and relative values (as percentages changes relative to baseline values). As will be seen, there were statistically significant improvements in seven out of the ten indicators, with changes of: − 4.23% (95% CI: − 5.26% to − 3.21%) in total DID prescribed; − 6.51% (95% CI: − 7.92 to − 5.22) in the use of penicillins; − 3.89% (95% CI: − 6.18 to − 1.65) in the use of cephalosporins; and − 3.45% (95% CI: − 5.23 to − 1.70) in the use of macrolides, lincosamides, and streptogramins.

**Table 3:** Impact of the intervention on the percentage reduction in each of the quality indicators across follow-up (median 19 months)

ESAC Quality Indicators [25]	Absolute Effect of intervention (95% CI)*	% reduction in intervention group relative to control group (95% CI)*	***p***-value*
1. DID of antibiotics for systemic use	−0.63 (− 0.78, − 0.48)	− 4.23 (− 5.26 to − 3.21)	< 0.0001
2. DID of penicillins	−0.52 (− 0.63, − 0.42)	−6.51 (− 7.92 to − 5.22)	< 0.0001
3. DID of cephalosporins	−0.07 (− 0.11, − 0.03)	− 3.89 (− 6.18 to − 1.65)	0.0002
4. DID of macrolides, lincosamides and streptogramins	−0.06 (− 0.09, − 0.03)	−3.45 (− 5.23 to − 1.70)	< 0.0001
5. DID of quinolones	−0.01 (− 0.05, 0.02)	−0.47 (− 2.37 to 0.93)	0.3736
6. PCT of beta-lactamase-sensitive penicillins^a^	−0.01 (− 0.06 to 0.05)	− 2.38 (− 15 to 11.63)	0.8223
7. PCT of combinations of penicillins with beta-lactamase inhibitors ^a^	−1.09 (− 1.42, − 0.76)	− 2.79 (− 3.65 to − 1.94)	< 0.0001
8. PCT of 3^rd^- and 4^th^-generationcephalosporins ^a^	−0.24 (− 0.36, − 0.12)	−5.69 (− 8.63 to − 2.80)	0.0001
9. PCT of quinolones	0.13 (− 0.06 to 0.33)	0.91 (− 0.42 to 2.30)	0.1709
10. Ratio of consumption of broad- to narrow-spectrum penicillins, cephalosporins and macrolides	- 5.37 (−8.23 to − 2.51)	−8.97 (− 13.99 to − 4.12)	0.0002

*Abbreviations*. *PCT* Percentage, *DID* Defined daily doses per 1000 inhabitants per day, *ESAC* European Surveillance of Antimicrobial Consumption

* Calculated from a mixed-effects model. The estimated value for intervention measures is an interaction between the variable “group” (intervention vs. control) and the variable “period” (post-intervention vs. baseline), adjusted for the secular trend of the indicator. The models were adjusted for the covariates of Table 1 and the seasonality. The percentage reduction was calculated by using the baseline values of the control group as reference values

^a^ Percentage of total consumption of antibiotics for systemic use in DID

Other noteworthy effects were changes in the percentage of 3^rd_^ and 4^th_^ generation cephalosporins of − 5.69% (95% CI: − 8.63 to − 2.80) with respect to total antibiotic consumption; and − 8.97% (95% CI: − 13.99 to − 4.12) with respect to the ratio of broad-spectrum antibiotics.

The power analysis of the total DID prescribed model yielded a statistical power of over 95%.

### Costs

Table 4 shows the reduction in costs, attributable to the intervention, in the intervention group across follow-up, with a breakdown by total costs (sum of the NHS and the population as a whole) and those of the NHS alone. Total antibiotic cost savings attributable to the intervention were 4.33, and 2.88% for the NHS. This reduction amounted to overall mean savings on antibiotic prescriptions per intervention-group physician of €573, and €311 for the Spanish NHS in Galicia. Total antibiotic cost savings per 1000 inhabitants attributable to the intervention were 4.46, and 2.43% for the NHS.

**Table 4:** Impact of the intervention on the reduction in cost (in euro) of antibiotics across follow-up (median 19 months)^a^

	% change in cost^a^			Changes in cost (in euro) across follow-up, attributable to the intervention ^b^	
	%	95%CI	***p***-value	€	95%CI
**Reduction in absolute direct costs attributable to the intervention**					
Costs for the Spanish NHS in Galicia	−2.88	−3.97 to − 1.8	< 0.0001	− 378,061	− 518,186 to − 237,936
Total costs	−4.33	−5.38 to − 3.29	< 0.0001	− 697,381	− 861,794 to − 533,216
**Reduction in direct costs per physician, attributable to the intervention**					
Cost for the Spanish NHS in Galicia	−2.88	− 3.97 to −1.8	< 0.0001	−311	− 426 to − 196
Total costs	−4.33	− 5.38 to − 3.29	< 0.0001	− 573	− 708 to − 438
**Reduction in direct costs per 1000 inhabitants, attributable to the intervention**					
Cost for the Spanish NHS in Galicia (1000 inhabitants)	−2.43	−3.55 to − 1.33	< 0.0001	− 205.8	− 298.5 to − 113.1
Total costs (1000 inhabitants)	− 4.46	− 5.54 to − 3.4	< 0.0001	− 464.7	− 573 to − 356.4

^a^ Calculated from a mixed-effects model. The estimated value for intervention measures is an interaction between the variable “group” (intervention vs. control) and the variable “period” (post-intervention vs. baseline), adjusted for secular trend of the indicator

^b^ Calculated by taking the total cost of antibiotics for the control group at baseline as reference

Table 5 gives a breakdown of all costs incurred in designing and implementing the intervention. The mean cost per intervention group physician (by intention-to-treat) was €86.96. Expressed in terms of ROI, every €1 invested in the intervention brought €5.59 in total return, and €2.57 to the NHS.

**Table 5:** Costs of intervention aimed at reducing antibiotic prescribing in primary care

Activity	Units	Cost per unit (€)	Cost (€)
*Identification of knowledge and attitudes*^a^			
Staff ^b^	2 months	2458.33	4916.66
Administrative support		Various	1599
Materials: letters, questionnaires, envelopes, postage stamps	4780	Various	4842.45
*Intervention study*			
*On-line course*			
Staff	1 month	2458.33	2458.33
On-line course (website programming and maintenance)	1	10,030	10,030
On-line course (contents)	1	2000	2000
Materials: overhead projector for presentations	1	500	500
*Outreach visit*			
Staff ^a^	6 months	2458.33	14,750
Travel and per diem expenses	Various	Various	3133.11
Telephone	6	20	120
Materials: leaflets for patients in health centre waiting rooms	45,000	0.0226	1017
*Costs for the participants*			
Physicians (sessions: 1 h)	409	23	9407
Physicians (course: 10 h)	222	230	51,060
**Total cost of intervention group** **(*n* = 1217)**	1217	–	105,833.55
**Mean cost per physician (by intention-to-treat)**	–	–	86.96

^a^ Evaluation of physicians’ knowledge and attitudes prior to the intervention (cohort study)^14^

^b^ University staff salary for a pharmacist. Includes national insurance, etc.

## Discussion

The results of this large-sized, pragmatic, cluster-randomised trial indicate that a multifaceted intervention targeting primary care physicians achieves a significant reduction in overall antibiotic prescribing and in the proportion of prescriptions of broad-spectrum antibiotics. Moreover, to our knowledge this is the first rigorously designed intervention to provide evidence of important direct cost savings, with an ROI of €5.59 (€2.57 for the Spanish NHS in Galicia), over a period of more than one and a half years. One possible reason for our success is that the low-cost intervention was designed on the basis of gaps in knowledge and attitudes previously shown to be linked to inappropriate prescription in the same target population.

### Magnitude of the effect

The magnitude of the effect found for total antibiotic prescribing, a reduction 4.23% (95% CI: 3.21 to 5.26%), is in line with other studies which report improvements of 4 to 12% attributable to interventions to improve antibiotic prescribing [31–37]. Some of these studies show a higher percentage because they are restricted to a specific indication. We feel that the magnitude of this effect is relevant from a public health standpoint, [34] especially bearing in mind that it is *underestimated,* owing to three factors: (1) The intervention was solely targeted at improving treatment of ARIs, which only account for 20–40% of total antibiotic prescriptions, [8–10] nonetheless, the effect of the intervention was assessed as *total antibiotics* prescribed and was thus underestimated; (2) Possible cross contamination between the intervention and control groups may also have led to underestimation of the impact of the intervention; and (3) Finally, the *study’s pragmatic nature,* which entails performing the statistical analysis by *intention-to-treat* and thus including all the physicians in the intervention group, regardless of whether or not they participated in the activities undertaken. While this avoids post-randomisation self-selection bias, it underestimates the effect of the intervention. Hence, we feel that the effects found are relevant, despite the relatively low percentage participation. Implementation of these types of interventions in a health system could be accompanied by economic or professional incentives for those who participated, thereby potentially ensuring that the effects might be even greater.

### Type of intervention

In the case of an intervention as low-cost as ours, its effectiveness is due to the fact that the intervention was expressly designed to address *gaps* in knowledge and *attitudes* which had been previously identified as being associated with inappropriate prescription in the same target population, and thereby permitted specific, concrete and more effective messages to be developed [14, 38]. The effectiveness of these types of educational interventions designed on the basis of previously identified gaps, [39, 40] has also been seen in a recent trial in Portugal [41]. We do not know to what extent these gaps exist in other countries and in other environments, though the differences observed between antibiotic prescription figures would lead one to assume that there are aspects of knowledge and attitudes which might differ across care settings. We therefore feel that, before setting out to design an educational intervention, a key factor should be to ascertain the prior knowledge and attitudes of the health professionals at whom it is to be targeted, so as to be able to focus the main thrust of the intervention on changing those facets of knowledge and attitudes that are more closely associated with inappropriate antibiotic prescribing. The intervention was designed to be as interactive as possible. We believe that the format itself would be applicable to other environments, especially due to its economic return. Furthermore, the messages were delivered via a group outreach visit, something that may in itself enhance effectiveness [30].

This is a *multifaceted, low-cost intervention*, with no need for highly qualified professionals to implement it or substantial sums of money to publicise it, which brings it closer to the real possibilities of healthcare systems. Furthermore, no financial incentives were used, something that might have increased physicians’ motivation but would have greatly increased the costs of the intervention (e.g., $1200 per physician in the case of Meeker et al. [34]) and impaired its applicability vis-à-vis public health systems.

### Relevance

Our study yielded relevant results in these indicators, obtaining a reduction in the consumption ratio of *broad*- to narrow-spectrum antibiotics of a magnitude of − 8.97% (95% CI: − 13.99% to − 4.14%), which is particularly significant, given the greater risk of resistance in the case of broad-spectrum antibiotics.

Furthermore, we see this intervention as being especially relevant from a public health point of view when analysed from a cost perspective, i.e., it results in a positive cost-benefit ratio, in that an investment of €105,834 brought direct savings of €378,061 for the Spanish NHS in Galicia. In other words, for every euro invested in the intervention, the Spanish NHS in Galicia realised an ROI of €2.57, which rises to a total ROI of €5.59 if the savings on antibiotic prescriptions for patients are also taken into account.

To calculate these returns on investment only direct benefits are borne in mind, without taking into account of the benefits associated with a decrease in the use of antibiotics, such as the reduction in adverse reactions, [42] hospitalisation costs, and/or costs of second-line inpatient antibiotic use [43]. A recent study undertaken in the USA estimates that the societal cost of antibiotic resistance increases antibiotic costs by 65% of the direct antibiotic costs [43]. While we do not know to what extent these US cost estimates are applicable to our study, it can nevertheless be assumed that, if all the social benefits were borne in mind, the social ROI of our intervention would be notably higher than that calculated on the basis of direct benefits. The above may serve to convince policymakers of the need to implement these types of interventions because, aside from improving prescribing, they would also save costs over a relatively short time horizon (19 months in our case).

### Strengths and limitations of this study

Our trial displays a number of strengths. The use of a *control group* served to control other potential sources of bias, such as seasonal variation or an effect of external interventions (such as changes in payment for prescribed medicines since April 2012, or the impacts of Spanish government and EU campaigns). By being *randomised*, our trial avoids potential selection bias; and a *cluster-based* distribution reduces the risk of cross-contamination between groups, though it raises the risk of groups becoming unbalanced by baseline values, particularly in cases, such as our study, with a small number of clusters [44]. We eliminated this effect in the statistical analysis, by: (1) adjusting for the *baseline values* of the dependent variables, by comparing the “before-and-after” changes in the intervention group against those in the control group, [19] and (2) adjusting for the covariates (baseline characteristics of intervention and control group) and seasonality.

A further strength of our study is that our data source only records prescriptions which have been collected by the patient from the pharmacy (i.e., dispensed). This is important, since it ensures that the database contains no record of delayed prescriptions which have not been dispensed by pharmacies.

Furthermore, the use of ESAC quality indicators as dependent variables prevents opportunistic selection of response variables, provides an acceptable measure to assess antibiotics use, and facilitates comparisons [25].

Our study possesses a series of limitations. First, the effect of the intervention was assessed solely on the basis of antibiotic ***prescription data,*** without taking into account the indications for which the drugs were prescribed. This means that we assessed the effect on the total of prescribed antibiotics (and not solely on prescriptions for diagnosis of ARI). However, the advantage of not considering the indications is that it minimises diagnostic shift, [27] in view of the fact that physicians who consider it necessary to prescribe an antibiotic for a specific patient, can choose an indication for which this antibiotic is recommended, even though the clinical indication in question is not the one presented by the patient at that particular point in time.

Second, the number of clusters was small (determined by the number of first-level hospitals in the study area), albeit within the acceptable range [45]. Moreover, we used Satterthwaite’s method to correct the degrees of freedom associated with the *p* values. Owing to the small number of clusters, the random distribution of the groups was not balanced by the basaline characteristics of the physicians and centres (see Table 1). However, this imbalance was addressed by adjusting for variables that were unequally distributed, as well as adjusting for baseline differences in the dependent variables. The other consideration is that, if any cross-contamination between groups occurred, the analysis would have been biased toward a null effect. In view of the fact that there were significant differences between groups, the true effect was underestimated, and would be probably stronger.

Third, should there be differences between the size of physicians’ patient list which might affect outcomes, we feel that this would not be associated -positively or negatively- with the exposure. This could cause a non-differential misclassification in the outcome, which would lead to an underestimate of the effect (and in turn towards the null hypothesis). If, despite this potential underestimate, the exposure shows an effect, it can be assumed that the effect would be greater still.

Fourth, the effect found might be thought to be due, wholly or in part, to the Hawthorne effect, caused by the control-group physicians’ ignorance of their participation in the study. However, due (1) to the type of outcome, based on records rather than self-reported data, and (2) to the duration of follow-up, it is highly unlikely that the effects found can be attributed to this bias [46].

Fifth limitation is that a sub-analysis of the doctors who underwent the entire intervention cannot be performed because, for data-confidentiality reasons, they cannot be associated with their prescriptions. The multifaceted intervention was designed so that all activities were carried out simultaneously, and as a result we did not evaluate the individual impact of each. From a pragmatic point of view, however, what interests us is the global effect, and not the subgroup effects, which would also increase type I error [47].

## Conclusions

In the context of the current global emergency surrounding the problem of resistance, and accepting that no single intervention can suffice to solve the problem of antibiotic misuse, we believe that the only viable option is to improve antibiotic use through the sum of effects. The results of this study indicate that low-cost interventions based on the previously identified gaps can be effective and, in addition, have a positive cost-benefit relationship over a short time horizon, something that could be highly relevant for their application by healthcare systems. If these results are repeated in other settings, they could be a great benefit for the global Public Health.

## Supplementary Information

**Additional file 1.** Patient-support materials.

**Additional file 2.** Supplementary online course.

**Additional file 3.** Supplementary intervention.

**Additional file 4.** Presentation of outreach visit.

## Data Availability

The data sharing plans for the current study are unknown and will made available at a later date: the database include global antimicrobial prescriptions in the Health Care System of Galicia (NHS). The data was provided by the NHS of Galicia, so we need their authorization before sharing it.

## Abbreviations

ROI: Return on Investment
NHS: Spanish National Health Service
ESAC: European Surveillance of Antimicrobial Consumption
DDD: Defined Daily Doses
ARIs: Acute Respiratory Infections
LMMs: Linear Mixed Models
IQR: Interquartile range
SD: Standard deviation
DID: Defined Daily Doses per 1000 inhabitants
PCT: Percentage
