# Supplementary material for: Impact of a multifaceted intervention to improve antibiotic prescribing: a pragmatic cluster-randomised controlled trial
Source: Antimicrob Resist Infect Control. 2020 Dec 7;9:195. doi: 10.1186/s13756-020-00857-9 (PMC7722452; doi:10.1186/s13756-020-00857-9)
Supplement: Supplementary file 1 — Additional file 1. Patient-support materials. [file 13756_2020_857_MOESM1_ESM.pdf]

Recuerde

- **Los antibióticos no son eficaces contra los resfriados ni la gripe**
- **Tome los antibióticos de forma responsable y sólo cuando se los recete el médico**
- **Todos somos responsables de que los antibióticos sigan siendo eficaces**

USC UNIVERSIDADE DE SANTIAGO DE COMPOSTELA

ecdc EUROPEAN CENTRE FOR DISEASE PREVENTION AND CONTROL

Cuando los *necesite*, utilice los antibióticos de manera responsable

Cuando le receten antibióticos, siga las instrucciones del médico para minimizar el riesgo de desarrollar bacterias resistentes.

Si no sigue las instrucciones correctamente —si, por ejemplo, acorta la duración del tratamiento o toma una dosis menor o no toma los antibióticos en los momentos correctos que le haya prescrito el médico—, las bacterias pueden adquirir resistencia a los antibióticos.

Las bacterias resistentes pueden quedarse en su organismo y también pueden transmitirse a otras personas. Esto le expone a usted y expone a otros al riesgo de no responder a los antibióticos la próxima vez que los vuelva a necesitar.

- **Siga las instrucciones de su médico y tome los antibióticos cuándo y cómo él se lo indique**
- **No utilice los antibióticos que le sobren**
- **Pregunte a su médico o farmacéutico cómo debe tirar los antibióticos que le sobren**

Si desea más información sobre el uso responsable de los antibióticos, visite: http://antibiotic.ecdc.europa.eu

Dep. Legal: C 2864-2011

EUROPEAN ANTIBIOTIC AWARENESS DAY

A European Health Initiative

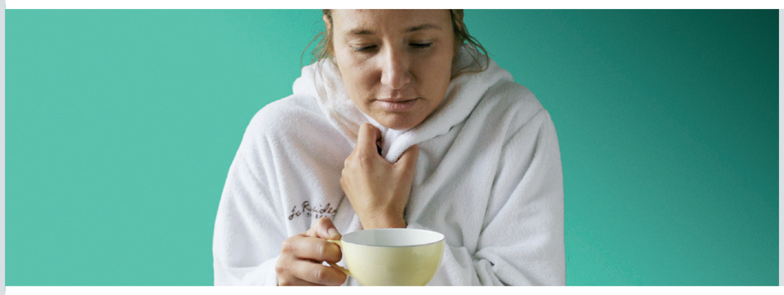

Uso responsable de los antibióticos

Contribuya a mantener la eficacia de los antibióticos

MINISTERIO DE CIENCIA E INNOVACIÓN

Instituto de Salud Carlos III

FONDO EUROPEO DE DESARROLLO REGIONAL
"Una manera de hacer Europa"

European Centre for Disease Prevention and Control (ECDC)
Tel: +46 (0)8 5860 1000
Email: EAAD@ecdc.europa.eu

USC UNIVERSIDADE DE SANTIAGO DE COMPOSTELA

ecdc EUROPEAN CENTRE FOR DISEASE PREVENTION AND CONTROL

Las bacterias resistentes a los antibióticos son un problema de salud cada vez más grave

Durante muchas décadas, los antibióticos han curado infecciones potencialmente mortales. Pero en años recientes, el uso incorrecto de los antibióticos ha provocado la aparición y propagación de bacterias que son resistentes a estos compuestos.

Cuando se contrae una infección causada por bacterias resistentes, los antibióticos no constituyen un tratamiento eficaz, y la enfermedad puede tardar más tiempo en curarse e incluso hacerse más grave.

El desarrollo de resistencia a los antibióticos es una amenaza grave para la salud pública, porque las bacterias resistentes pueden propagarse entre la población.

- **Todos somos responsables de que los antibióticos sigan siendo eficaces**
- **El uso responsable de antibióticos puede contribuir a frenar la aparición de bacterias resistentes**
- **Detener la aparición de bacterias resistentes hará que los antibióticos sigan siendo eficaces para las generaciones futuras**

Tome antibióticos sólo si es estrictamente necesario

Los antibióticos deben utilizarse sólo cuando sea necesario, porque el uso excesivo contribuye a la aparición de bacterias resistentes a estos medicamentos.

Tomar antibióticos cuando no son necesarios no sirve de nada. Los antibióticos NO son eficaces frente a las infecciones causadas por virus, como el resfriado común o la gripe. En la mayoría de los casos, el sistema inmunitario debe ser capaz de combatir las infecciones leves. Tomar antibióticos no favorece la recuperación ni evita la difusión de los virus a otras personas.

Sólo un médico puede establecer un diagnóstico correcto y decidir si su enfermedad requiere tratamiento antibiótico.

- **No tome antibióticos si no se los ha recetado un médico**
- **Si contrae un resfriado o una gripe y el médico le dice que no necesita antibióticos, cuídese para recuperarse**
- **Si los síntomas no mejoran o empeoran en el plazo indicado por el médico, vuelva a la consulta para que lo examine de nuevo**

EUROPEAN ANTIBIOTIC AWARENESS DAY

A European Health Initiative

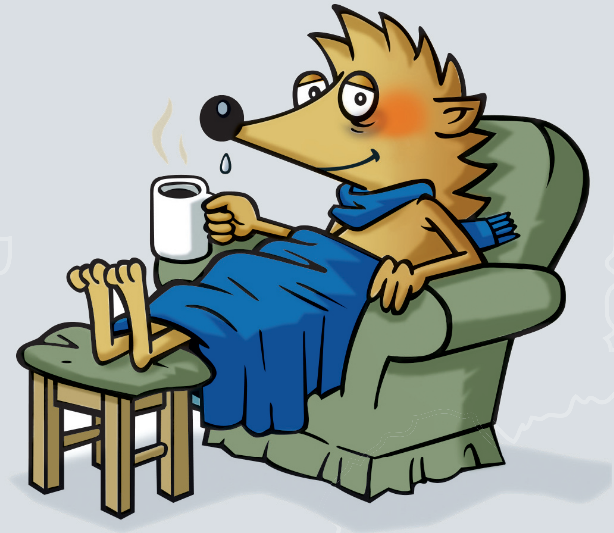

¿Resfriado? ¿Gripe?

Necesita cuidarse, no tomar antibióticos

¿Qué debe hacer si contrae un resfriado o una gripe y el médico no le receta antibióticos?

1. Descanse y cuídese para recuperarse
2. Beba mucho líquido para no deshidratarse
3. Pregunte al médico si hay algún tratamiento no antibiótico para aliviar síntomas como la nariz taponada, el dolor de garganta, la tos, etc.

Si los síntomas no mejoran o empeoran en el plazo indicado por el médico, vuelva a la consulta para que lo examine de nuevo.
