# Supplementary material for: Impact of a multifaceted intervention to improve antibiotic prescribing: a pragmatic cluster-randomised controlled trial
Source: Antimicrob Resist Infect Control. 2020 Dec 7;9:195. doi: 10.1186/s13756-020-00857-9 (PMC7722452; doi:10.1186/s13756-020-00857-9)
Supplement: Supplementary file 2 — Additional file 2. Supplementary online course. [file 13756_2020_857_MOESM2_ESM.pdf]

Appendix 2 – Supplementary Online Course: *appearance and example of running*

- Presentación
- Información General
- Metodología
- Casos Clínicos
- Algoritmos
- Bibliografía de apoyo
- Salir

Infecciones respiratorias en Atención Primaria: puesta al día

Objetivo

Actualización en el manejo de las enfermedades infecciosas respiratorias comunitarias, mediante la difusión de recomendaciones consensuadas sobre cómo utilizar los recursos diagnósticos y terapéuticos.

Justificación

En Atención Primaria se realiza más de un 80% de la prescripción de antibióticos, en su mayor parte empleados para el tratamiento de infecciones respiratorias que generalmente están causadas por virus. A la hora de orientar el proceso infeccioso, debemos llevar a cabo una anamnesis y exploración física orientadas al caso, ayudándonos de métodos de diagnóstico rápido, si están a nuestro alcance, y decidiendo si es necesario prescribir un antibiótico para el microorganismo responsable de la infección. Si optamos por utilizar antibiótico, debemos considerar no sólo los beneficios esperados sino también los efectos no deseados, que afectan tanto al individuo como a su entorno. La selección del fármaco dependerá de si se conoce el patógeno o no; este último caso es el habitual en nuestro medio, teniendo que optar frecuentemente por emplear tratamientos empíricos, en función de otras características como la fuente de infección, las circunstancias del paciente y la epidemiología local, en el caso de que sea conocida.

El taller que se ha diseñado pretende ser de utilidad en la orientación diagnóstica y preciso en la elección del antibiótico, contribuyendo también de esta forma en la mejora de un importante problema de salúd pública: las resistencias bacterianas. España es uno de los países con mayores tasas de resistencia y ésta se asocia con una mayor morbi-mortalidad, demanda sanitaria, gasto sanitario y deterioro de la eficacia del tratamiento de futuros pacientes. El consumo de antibióticos no es el único factor pero constituye un aspecto clave. Debemos prescribir menos antibióticos y utilizar aquellos con espectro más reducido.

Basado en el Manual de enfermedades infecciosas en Atención Primaria, 3ª ed. Barcelona: semfyc ediciones, 2010

semFYC
Sociedad Española de Medicina de Familia y Comunitaria

Autores

Jose Mª Molero García

Especialista en Medicina Familiar y Comunitaria.
Coordinador del capítulo Infecciones del aparato respiratorio superior de la 3ª Edición del Manual de enfermedades infecciosas en Atención Primaria (2010).
Miembro del Grupo de Trabajo de Enfermedades Infecciosas de la semFYC. Sociedad Madrileña de Medicina Familiar y Comunitaria (SBMFiC).
Con la colaboración de **Jesús Redondo Sánchez del Grupo de enfermedades infecciosas de la SoMaMFyC**.

Jose Mª Cots Yago

Especialista en Medicina Familiar y Comunitaria.
Coordinador del capítulo Infecciones del aparato respiratorio inferior de la 3ª Edición del Manual de enfermedades infecciosas en Atención Primaria (2010).
Coordinador del Grupo de Trabajo de enfermedades infecciosas de la semFYC. Sociedad Catalana de Medicina Familiar y Comunitaria (CAMFiC).
Con la colaboración de Coro Sánchez Hernández. Grupo de Trabajo de enfermedades infecciosas de la semFYC.

Tutores

Jose Mª Molero García. Sociedad Madrileña de Medicina Familiar y Comunitaria (SBMFiC).
Jose Mª Cots Yago. Sociedad Catalana de Medicina Familiar y Comunitaria (CAMFiC).
Mª José Monedero Mira. Sociedad Valenciana de Medicina Familiar y Comunitaria (SVMFiC).
Javier Arranz Izquierdo. Sociedad Balear de Medicina Familiar y Comunitaria (SBMFiC).
Mª Luisa Morató Agustí. Sociedad Catalana de Medicina Familiar y Comunitaria (CAMFiC).
Coro Sánchez Hernández. Sociedad Gallega de Medicina Familiar y Comunitaria (AGAMFEC).
Con la colaboración como **consultor externo** de Abel Pallarés. Servicio de Neumología. Complejo Hospitalario de Pontevedra.

Presentación
Información General
Autores
Tutores
Coordinación
Acreditación
Promotores
Metodología
Casos Clínicos
Algoritmos
Bibliografía de apoyo
Salir

Coordinación

Adolfo Figueiras Guzmán. Medicina Preventiva. Universidad de Santiago de Compostela.
Coro Sánchez Hernández. Médico de Familia. Sociedad Gallega de Medicina Familiar y Comunitaria (AGAMFEC).

Presentación
Información General
Autores
Tutores
Coordinación
Acreditación
Promotores
Metodología
Casos Clínicos
Algoritmos
Bibliografía de apoyo
Salir

Acreditación

Actividad acreditada por la Comisión Autonómica de Formación Continuada de la Comunidad Autónoma de Galicia con 2,3 créditos.

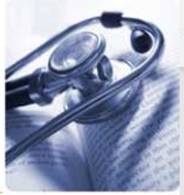

Promotores

Curso financiado por el Instituto de Salud Carlos III, perteneciente al proyecto PI09/90609, desarrollado en la Universidad de Santiago de Compostela con la colaboración del GdT de enfermedades infecciosas de la semFYC.

Manual del usuario

El curso se desarrollará mediante metodología on-line a través de un campus virtual de aprendizaje desarrollado por la empresa Contacnova S. L., empresa colaboradora habitual de la Universidad de Santiago de Compostela. Además del diseño se encargará de dar soporte técnico a lo largo del curso.
Se facilitará a cada alumno una clave de acceso y una dirección de correo donde podrá contactar con los tutores para formular sus dudas.
El plazo para la realización del curso completo será de 4 semanas.

Para acceder al contenido del curso necesita introducir su dirección de e-mail a la vez que una clave autorizada por el profesorado que le remitirá a su correo electrónico.

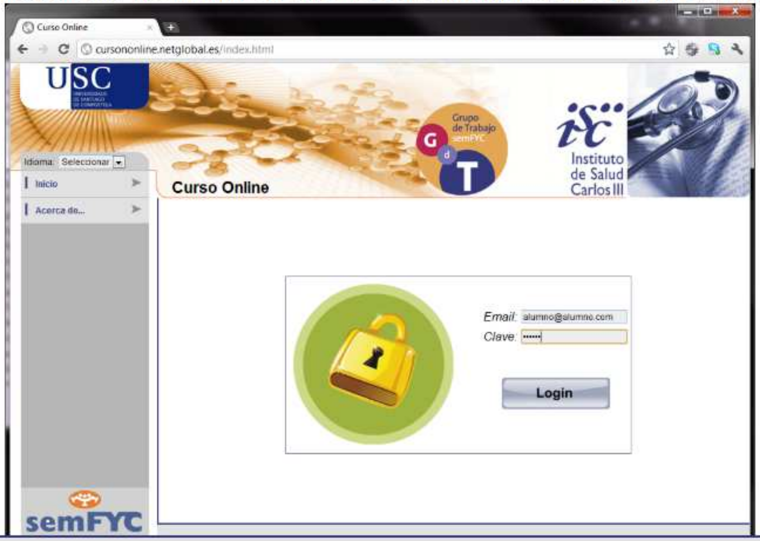

Una vez introducidos y confirmados nuestros datos correctamente, accedemos automáticamente en la aplicación del curso online sobre enfermedades infeccionas del aparato respiratorio en Atención Primaria, donde nos aparecerá la siguiente pantalla:

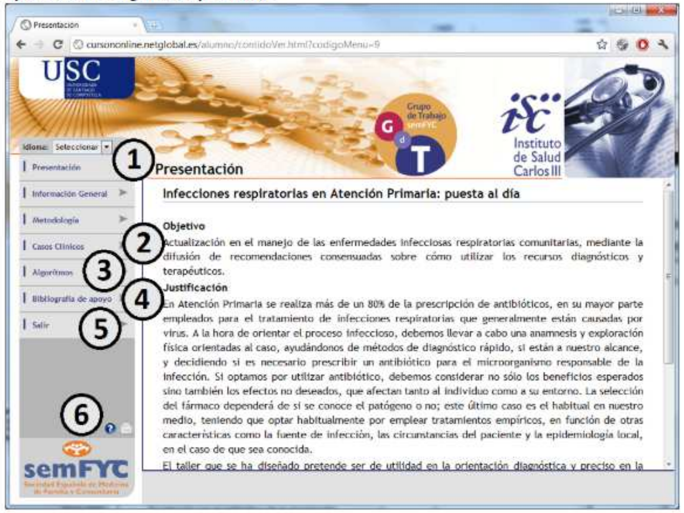

1. Menú de información general con una pequeña presentación sobre el curso, autores, tutores, coordinación, acreditación, promotores, metodología y manual de usuario.
2. Acceso a la información del curso organizado en 5 casos clínicos.
3. Acceso a los Algoritmos visuales y Algoritmos en Software que nos facilitaran el diagnóstico de las enfermedades respiratorias según el cuadro del caso clínico a evaluar. En un inicio los algoritmos están ocultos e inaccesibles hasta que los casos clínicos se vayan completando y superando con éxito.
4. Botón de acceso a Bibliografía de apoyo
5. Botón de salir de la aplicación
6. Botón de envío de mensaje a los tutores o a soporte técnico para resolver posibles dudas sobre los casos clínicos.

Si accedemos al primer caso clínico tendremos una serie de iconos y funcionalidades nuevas:

Si accedemos al primer caso clínico tendremos una serie de iconos y funcionalidades nuevas:

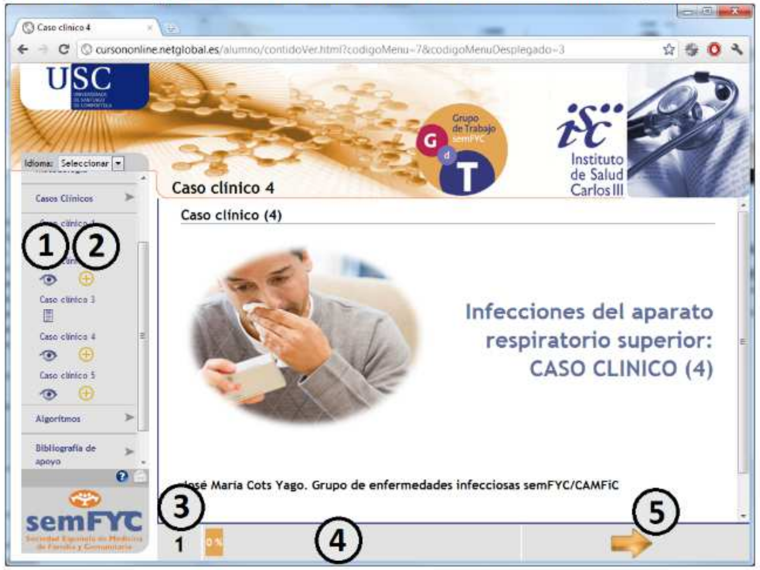

1. Este icono nos permite visualizar el caso clínico y retomarlo en la última diapositiva que se visualizó.
2. Este icono nos permite visualizar en cualquier momento del curso y en una ventana emergente de nuestro navegador los datos del caso clínico.
3. Nº de diapositiva en la que nos encontramos
4. Barra de progreso del caso clínico actual
5. Botones de navegación en el caso clínico.

A medida que avanzamos el caso clínico, la aplicación nos pondrá a prueba con una serie de preguntas.

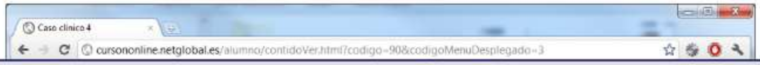

Presentación
Información General
Metodología
Manual del usuario
Sistema de Evaluación
Casos Clínicos
Algoritmos
Bibliografía de apoyo
Salir

5. Botones de navegación en el caso clínico.

A medida que avanzamos el caso clínico, la aplicación nos pondrá a prueba con una serie de preguntas.

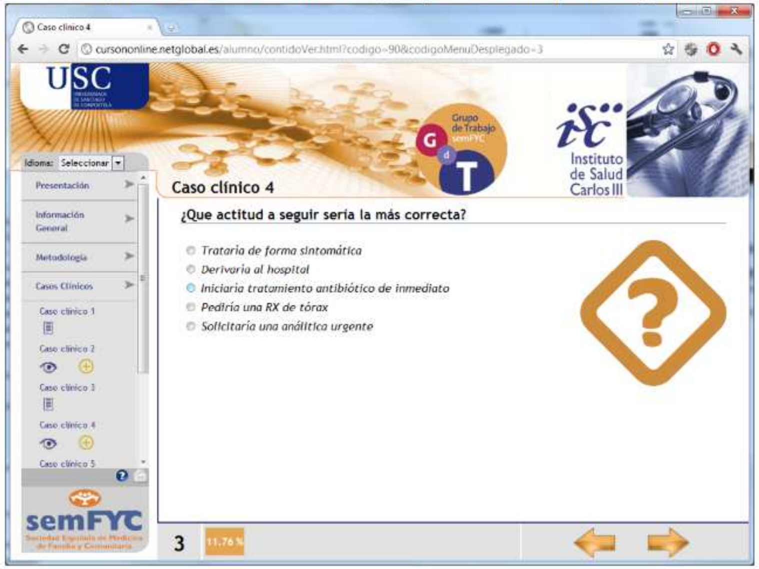

Debemos elegir la respuesta que consideremos más acertada. Al final del caso clínico y una vez adquirido los conocimientos necesarios, la aplicación nos permitirá repasar las respuestas dadas durante el transcurso del curso y podremos corregir aquellas que consideremos erróneas.

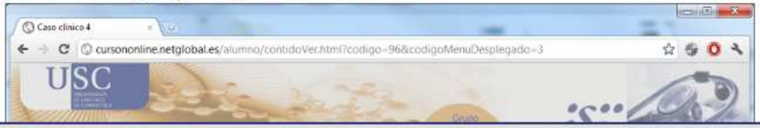

Presentación
Información General
Metodología
Manual del usuario
Sistema de Evaluación
Casos Clínicos
Algoritmos
Bibliografía de apoyo
Salir

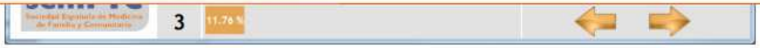

Debemos elegir la respuesta que consideremos más acertada. Al final del caso clínico y una vez adquirido los conocimientos necesarios, la aplicación nos permitirá repasar las respuestas dadas durante el transcurso del curso y podremos corregir aquellas que consideremos erróneas.

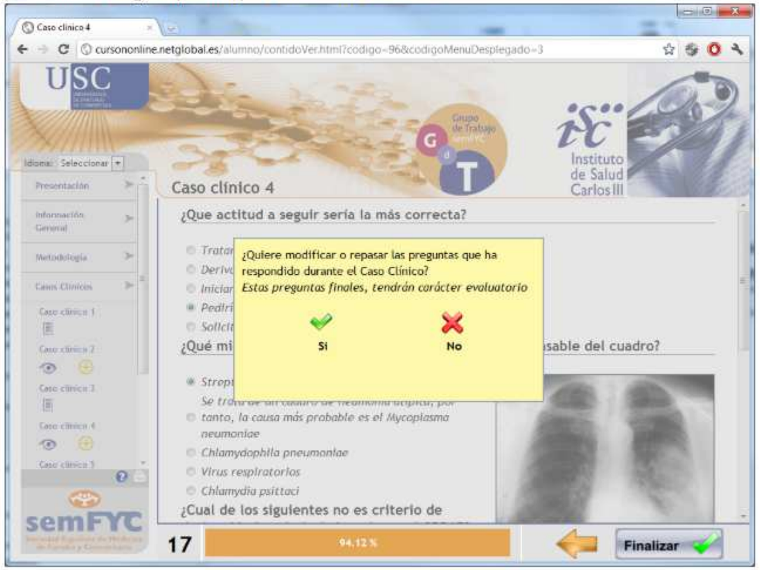

En el caso de que el caso clínico no fuese superado debemos reiniciarlo y no se desbloqueará el algoritmo correspondiente.

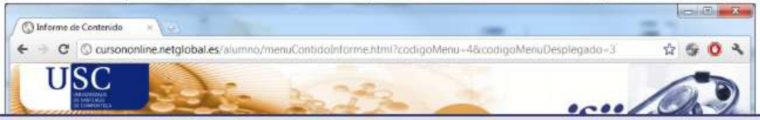

Sociedad Española de Medicina de Familia y Comunitaria

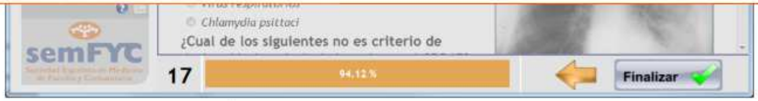

En el caso de que el caso clínico no fuese superado debemos reiniciarlo y no se desbloqueará el algoritmo correspondiente.

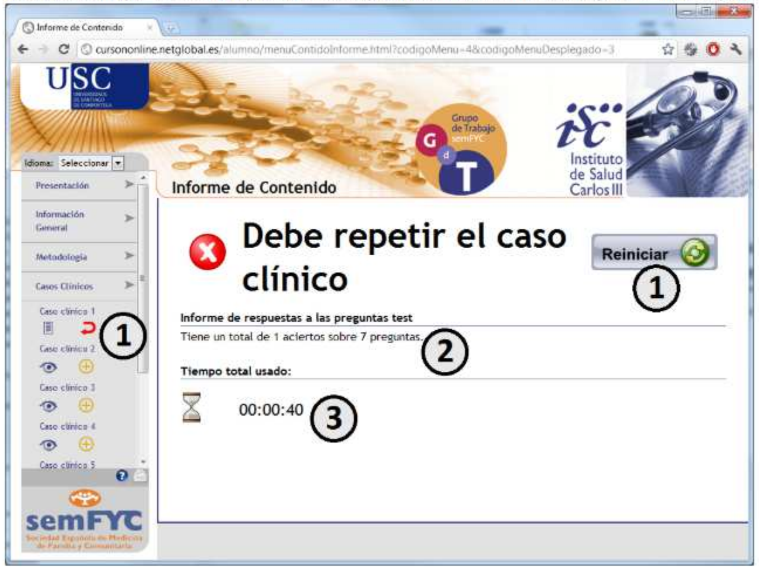

1. Botones para reinicializar el curso desde un principio
2. Informe del nº de aciertos sobre el total de preguntas
3. Tiempo de consulta total de las diapositivas.

Presentación
Información General
Metodología
Manual del usuario
Sistema de Evaluación
Casos Clínicos
Algoritmos
Bibliografía de apoyo
Salir

1. Botones para reinicializar el curso desde un principio
2. Informe del nº de aciertos sobre el total de preguntas
3. Tiempo de consulta total de las diapositivas.

Una vez superado el caso clínico se mostrará la siguiente pantalla que detallamos a continuación:

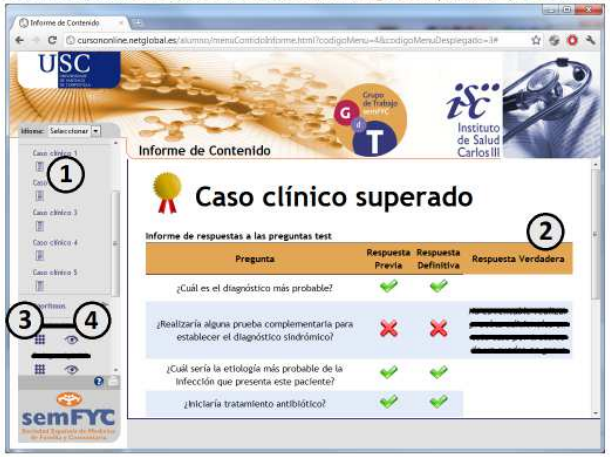

1. Botón para visualizar el informe del caso clínico
2. Informe del caso clínico con las respuestas correctas e incorrectas contestadas durante la realización del curso y finalmente la respuesta definitiva dada. Si una pregunta fue contestada incorrectamente en el test final, la respuesta verdadera aparecerá en una 4ª columna.
3. Como el caso clínico fue superado con éxito, queda accesible el algoritmo correspondiente. Así con el siguiente botón podremos visualizar el algoritmo que nos ayudará en nuestro diagnóstico.
4. Con este botón accedemos al algoritmo por software, que nos irá realizando una serie de preguntas y finalmente nos aconsejará en nuestra toma de decisión a la hora de diagnosticar.

Presentación
Información General
Metodología
Manual del usuario
Sistema de Evaluación
Casos Clínicos
Algorítmos
Bibliografía de apoyo
Salir

Sistema de Evaluación

El curso está dividido en 5 casos clínicos que servirán de introducción a los conceptos teóricos. Se realizará una prueba de evaluación al final con preguntas tipo test, en la que el alumno tendrá la oportunidad de valorar su evolución contrastando sus propias respuestas antes y después de realizar los casos.

Requerimientos exigidos para la acreditación

Haber superado en la prueba de evaluación final el 50 % de aciertos.

Manejo de la rinosinusitis bacteriana aguda

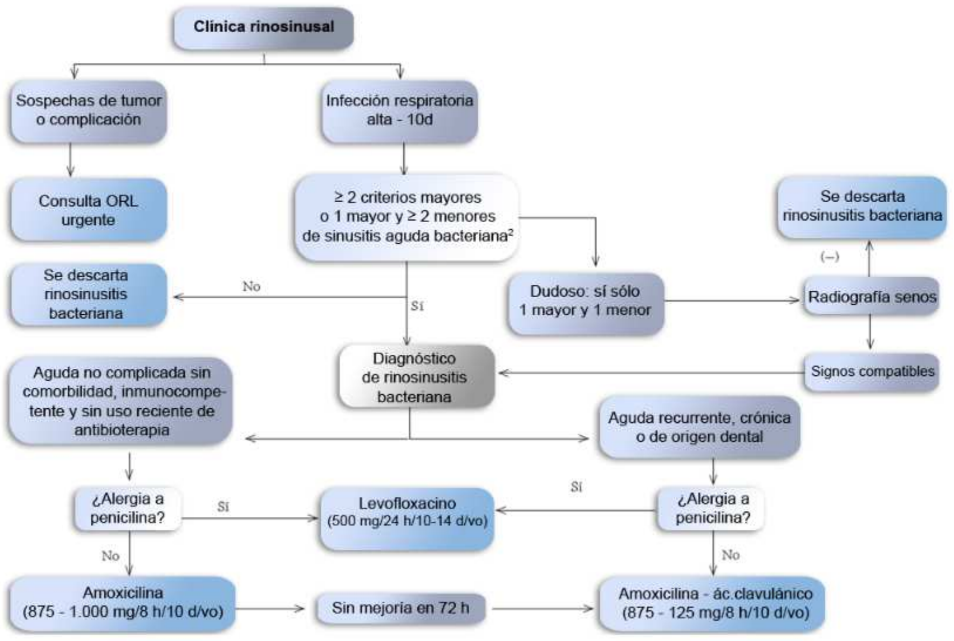

[2] *Ver diapositiva de diagnóstico clínico*

Manual de enfermedades infecciosas en Atención Primaria, 3ª ed. Barcelona: semfyc ediciones, 2010

Algoritmo diagnóstico en faringitis

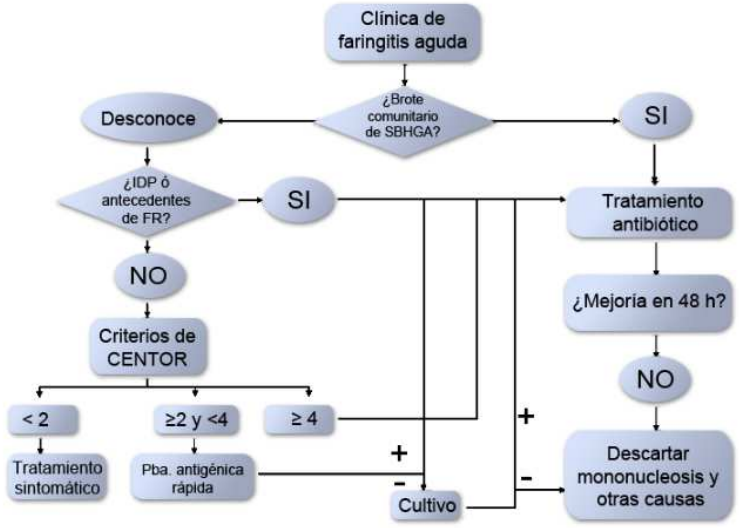

SBHGA: streptococcus betahemolítico grupo a
FR: fiebre reumática
IDP: Inmunodepresión

Manual de enfermedades infecciosas en Atención Primaria, 3ª ed. Barcelona: semfyc ediciones, 2010

Algoritmo de actuación ante una bronquitis aguda

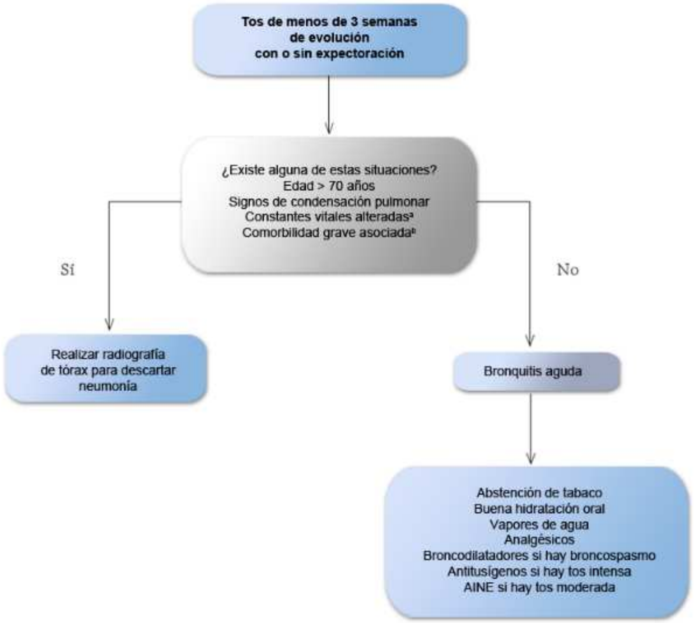

a. Temperatura axilar > 39 ºC; frecuencia respiratoria >24/min, o bien frecuencia cardíaca >100/min.
b. Accidente cerebrovascular, EPOC, insuficiencia renal, cirrosis, inmunodepresión, neoplasia, o bien insuficiencia cardiaca
AINE: antiinflamatorios no esteroideos; EPOC: enfermedad pulmonar obstructiva crónica.

semFYC Sociedad Española de Medicina de Familia y Comunitaria

Algoritmo de abordaje de paciente con probable neumonía

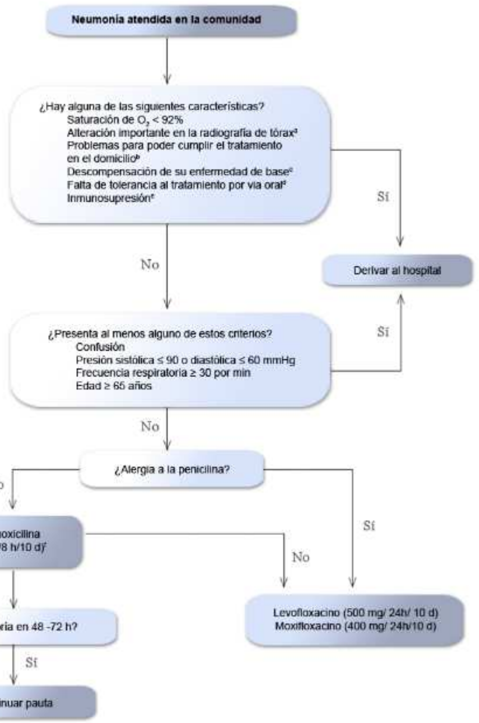

[a] Hay que disponer de una radiografía de tórax para confirmar el diagnóstico y evaluar la gravedad de la enfermedad cuando se presenta cualquiera de las siguientes alteraciones: infiltrado alveolar en más de un lóbulo o bilateral, derrame pleural o cavitación pulmonar.

[b] Sociopatía o problemas psiquiátricos, adicción a drogas y alcohol o un entorno familiar no adecuado.

[c] Hay que prestar especial atención a la presencia de insuficiencia renal, cirrosis hepática, insuficiencia cardíaca, enfermedad pulomonar obstructiva crónica, accidente cerebrovascular y diabetes mellitus.

[d] Presencia de náuseas y vómitos, gastrectomía, postcirugía o diarrea franca.

[e] Infección crónica por virus de la inmunodeficiencia adquirida, trasplantados, neutropénicos o pacientes que reciben tratamiento inmunosupresor.

[f] En pacientes mayores de 65 años, debería utilizarse la asociación de amoxicilina y ácido clavulánico (2.000 - 125 mg/ 12 h/ 10 d) para cubrir *Haemophilus influenzae*.

Algoritmo de actuación ante una agudización en un paciente EPOC

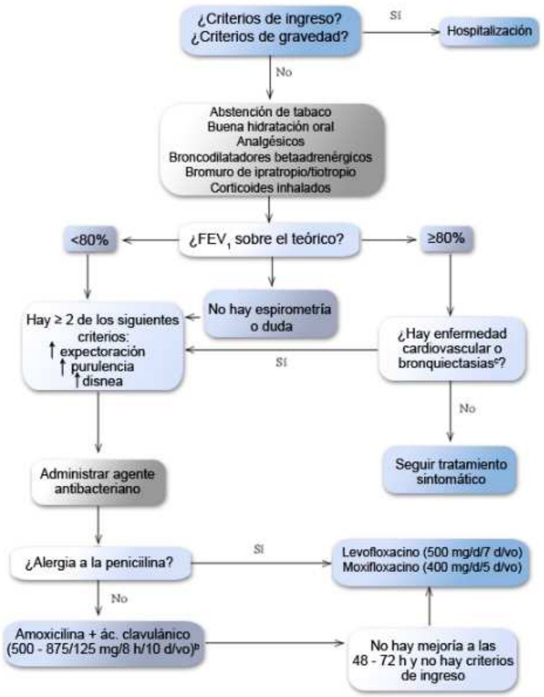

[a]Obnubilación, taquipnea (>35rpm), insuficiencia respiratoria, *cor pulmonale*, *sociopatía*. Cuando hay comorbilidad asociada (cirrosis hepática, insuficiencia hepática, diabetes insulino-dependiente, insuficiencia cardíaca, cardiopatía isquémica), puede instarurarse tratamiento de forma ambularotia y controlar estrictamente el paciente.

[b]En caso de intolerancia al ácido clavulánico puede utilizarse una cefalosporina.

[c]Algunos autores consideran también la edad avanzada (mayores de 65 años) y los pacientes con cuatro o más agudizaciones al año.

- Presentación
- Información General
- Metodología
- Casos Clínicos
- Algoritmos
- Bibliografía de apoyo
- Bibliografía de infecciones respiratorias altas
- Bibliografía de infecciones respiratorias bajas
- Salir

semFYC Sociedad Española de Medicina de Familia y Comunitaria

Bibliografía de infecciones respiratorias altas

- Tomás M, Ortega P, Mensa J, García JA, Barberan J. Diagnóstico y tratamiento de las rinosinusitis agudas. Segundo consenso. Rev Esp Quimioter 2008;21(1):45-59
- Arroll B, Kenealy T. Antibióticos para el resfriado común y la rinitis purulenta aguda (Revisión Cochrane traducida). En: La Biblioteca Cochrane Plus, número 3, 2008. Oxford, Update Software Ltd. Disponible en: http://www.update-software.com. (Traducida de The Cochrane Library, Issue . Chichester, UK: John Wiley & Sons, Ltd.).
- Young J, De Sutter A, Merenstein D, van Essen GA, Kaiser L, Varonen H, Williamson I, Bucher HC. Antibiotics for adults with clinically diagnosed acute rhinosinusitis: a meta-analysis of individual patient data.Lancet. 2008; 371(9616):908-14.
- Falagas ME, Giannopoulou KP, Vardakas KZ, Dimopoulos G, Karageorgopoulos DE. Comparison of antibiotics with placebo for treatment of acute sinusitis: a meta-analysis of randomised controlled trials. Lancet Infect Dis. 2008; 8(9):543-52
- Tomás M, Ortega P, Mensa J, García JA, Barberan J. Diagnóstico y tratamiento de las rinosinusitis agudas. Segundo consenso. Rev Esp Quimioter 2008;21(1):45-59
- Fokkens WJ, Lund VJ, Mullol J; European Position Paper on Rhinosinusitis and nasal Polips Group. European Position Paper on Rhinosinusitis and Nasal Polyps 2007. Rhinology 2007; 45(Suppl. 20):1 -139.
- Rosenfeld RM, Singer M, Jones S. Systematic review of antimicrobial therapy in patients with acute rhinosinusitis.Otolaryngol Head Neck Surg. 2007; 137(3 Suppl):S32-45.
- Del Mar CB, Glasziou PP, Spinks AB Antibióticos para la faringitis (Revisión Cochrane traducida). En: La Biblioteca Cochrane Plus, 2005 Número 2. Oxford: Update Software Ltd. Disponible a: http://www.update-software.com. (Traducida de The Cochrane Library, 2005 Issue 2. Chichester, UK: John Wiley & Sons, Ltd.).
- Maltezou HC, Tsagris V, Antoniadou A, Galani L, Douros C, Katsarolis I, et al. Evaluation of a rapid antigen detection test in the diagnosis of streptococcal pharyngitis in children and its impact on antibiotic prescription. J Antimicrob Chemother. 2008.
- Centor R, Witherspoon J, Dalton H, Brody C, Link K. The diagnosis of strep throat in adults in the emergency room. Med Decis Mak. 1981;1(3):239-46.1
- Snow V, Mottur-Pilson C, Cooper RJ, Hoffman JR; American Academy of Family Physicians; American College of Physicians-American Society of Internal Medicine; Centers for Disease Control. Principles of appropriate antibiotic use for acute pharyngitis in adults. Ann Intern Med. 2001 Mar 20;134(6):506-8.

Bibliografía de infecciones respiratorias bajas

- Smucny JJ, Becker LA, Glazier RH, McIsaac W. Are antibiotics effective treatment for acute bronchitis? J Fam Pract 1998;47:453-60.
- Coenen S, Van Royen P, Vermeire E, Hermann I, Denekens J. Antibiotics for coughing in general practice: a qualitative decision analysis. Fam Pract 2000;17:380-5.
- Nduba VN, Mwachari CW, Magaret AS, Park DR, Kigo A, Hooton TM, et al. Placebo found equivalent to amoxicillin for treatment of acute bronchitis in Nairobi, Kenya: a triple blind, randomized, equivalence trial. Thorax 2008;63:999-1005.
- Stott NC, West RR. Randomised controlled trial of antibiotics in patients with cough and purulent sputum. Br Med J 1976;2:556-9.
- Bent B, Saint S, Vittinghoff E, Grady D. Antibiotics in acute bronchitis: a meta-analysis. Am J Med 1999;107:62-7.
- Petersen I, Johnson AM, Islam A, Duckworth G, Livermore DM, Hayward AC. Protective effect of antibiotics against serious complications of common respiratory tract infections: retrospective cohort study with the UK General Practice Research Database. BMJ 2007;335:982.
- Spurling GK, Del Mar CB, Dooley L, Foxlee R. Delayed antibiotics for respiratory infections.Cochrane Database Syst Rev 2007;(3):CD004417.
- Smucny J, Fahey T, Becker L, Glazier R. Antibiotics for acute bronchitis. Cochrane Database Syst Rev 2004;(4):CD000245.
- Anthonisen NR, Manfreda J, Warren CPW, Hershfield ES, Harding GKM, Nelson NA. Antibiotic therapy in exacerbations of chronic obstructive pulmonary disease. Ann Intern Med 1987;106:196-204.
- Stockley RA, O'Brien C, Pye A, Hill SL. Relationship of sputum color to nature and outpatient management of acute exacerbations of COPD. Chest 2000;117:1638-45.
- Ball P. Epidemiology and treatment of chronic bronchitis and its exacerbations. Chest 1995;108:43S-52S.
- Saint S, Bent S, Vittinghoff E, Grady D. Antibiotics in chronic obstructive pulmonary disease exacerbations: a meta-analysis. JAMA 1995;273:957-60.
- Ram FS, Rodríguez-Roisin R, Granados-Navarrete A, García-Aymerich J, Barnes NC. Antibiotics for exacerbations of chronic obstructive pulmonary disease. Cochrane Database Syst Rev 2006;(2):CD004403.
- Miravitlles M, Monsó E, Mensa J, Aguarón J, Barberán J, Bárcena M, et al, Antimicrobial Treatment of Exacerbation in Chronic Obstructive Pulmonary Disease: 2007 Consensus Statement. Arch Bronconeumol 2008;44:100-8.
- Puhan MA, Vollenweider D, Latshang T, Steurer J, Steurer-Stey C. Exacerbations of chronic obstructive pulmonary disease: when are antibiotics indicated? A systematic review. Respir Res 2007;8:30.
- Rohde G, Wiethege A, Borg I, Kauth M, Bauer TT, Gillissen A, et al. Respiratory viruses in exacerbations of chronic obstructive pulmonary disease requiring hospitalisation: a case-control study. Thorax 2003;58:37-42.
- Lieberman D, Shmarkov O, Gelfer Y, Varsavsky R, Lieberman DV. Prevalemce and clinical significance of fever in acute exacerbations of chronic obstructive pulmonary disease. Eur J Clin Microbiol Infect Dis 2003;22:75-8.
- Fine MJ, Auble TE, Yealy DM, Hanusa BH, Weissfeld LA, Singer DE, et al. A prediction rule to identify low-risk patients with community-acquired pneumonia. N Engl J Med 1997;336: 243-50.
- Metlay JP, Fine MJ. Testing strategies in the initial management of patients with community-acquired pneumonia. Ann Intern Med 2003;138:109-18.
- Schuetz P, Koller M, Christ-Crain M, Steyerberg E, Stolz D, Müller C, et al. Predicting mortality with pneumonia severity scores: importance of model recalibration to local settings. Epidemiol Infect 2008;27:1-10.
- Lim WS, Van der Erden MM, Boersma WG, Karalus N, Town GI Lewis SA, et al. Defining community acquired pneumonia severity on presentation to hospital: an international derivation and validation study. Thorax 2003;58:337-82.
- Santos C, Llorente MA, Carandell E, Gutiérrez M, Riera M, Ramírez A et al. Lugar de atención, etiología y tratamiento de las neumonías adquiridas en la comunidad de Palma de Mallorca. Med Clín (Barc) 1998;110:290-4.
- Mandell LA, Wunderink RG, Anzueto A, Bartlett JG, Campbell GD, Dean NC, et al. Infectious Diseases Society of America/American Thoracic Society consensus guidelines on the management of community-acquired pneumonia in adults. Clin Infect Dis 2007;44(Suppl 2):S27-72.
- Lim WS. Severity assessment in community-acquired pneumonia: moving on. Thorax 2007;62:287-8.
- Almirall J, Bolíbar I, Torán P, Pera G, Boquet X, Balanzó X, Sauca G; Community-acquired pneumonia Maresme Study Group. Contribution of C-reactive protein to the diagnosis and assessment of severity of community-acquired pneumonia. Chest 2004;125:1335-42.
- Mills GD, Oehley MR, Arrol B. Effectiveness of betalactam antibiotics compared with antibiotics active against atypical pathogens in non-severe community acquired pneumonia: meta-analysis. BMJ 2005;330:456.
- Holm A, Nexoe J, Bistrup LA, Pedersen SS, Obel N, Nielsen LP, Petersen C. Aetiology and prediction of pneumonia in lower respiratory tract infection in primary care. Br J Gen Pract 2007;57:547-54.
- Falk G, Fahey T. C-reactive protein and community-acquired pneumonia in ambulatory care: systematic review of diagnostic accuracy studies. Fam Pract 2009;26:10-21.

Caso clínico 1

Caso clínico (1)

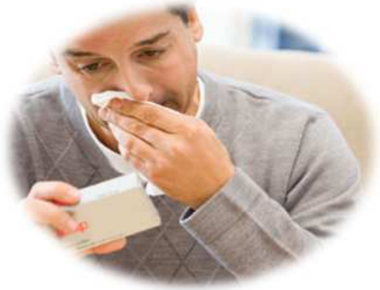

Infecciones del aparato respiratorio superior: CASO CLINICO (1)

José María Molero García. Grupo de enfermedades infecciosas semFYC/SoMaMFyC
Con la colaboración de Jesús Redondo Sánchez. Grupo de enfermedades infecciosas SoMaMFyC

Presentación
Información General
Metodología
Casos Clínicos
Caso clínico 1
Caso clínico 2
Caso clínico 3
Caso clínico 4
Caso clínico 5
Algoritmos
Bibliografía de apoyo
Salir

Caso clínico (1)

Varón de 35 años.

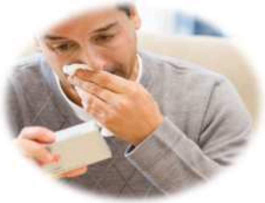

Antecedentes:
- Amigdalectomizado (hace 7 años). Fumador 10 cigarrillos/día (15 paquetes-año).
- Resfriados frecuentes en invierno (4 episodios /año).
- Intervenido de pólipo nasal hace 2 años.

Enfermedad actual:
- Cuadro de **"catarro nasal"** que arrastra desde hace **10 días** que no ha mejorado, incluso refiere encontrarse peor que en los primeros días.
- **Rinorrea amarillenta-verdosa en últimos 3 días** con obstrucción nasal.
- **Fiebre (38,5° C)** en las últimas 36 horas.
- **Cefalea frontal y en hemicara derecha** moderada que se controla parcialmente con analgesia.
- Tos seca irritativa desde el comienzo del cuadro, sin disnea.

Exploración:
- Afectación del estado general, Tª :38,6°C.
- FC: 72 lpm; FR: 17 rpm; Saturación O_2: 95%.
- Mucosa nasal congestionada, hiperémica.
- Secreción muco-purulenta en cavum.
- Otoscopia normal.
- Dolor a la presión maxilar derecho.

Presentación
Información General
Metodología
Casos Clínicos
Caso clínico 1
Caso clínico 2
Caso clínico 3
Caso clínico 4
Caso clínico 5
Algoritmos
Bibliografía de apoyo
Salir

Caso clínico 1

¿Cuál es el diagnóstico más probable?

- ○ *Rinitis aguda.*
- ○ *Bronquitis aguda*
- ◉ *Rinosinusitis aguda bacteriana.*
- ○ *Traqueitis.*
- ○ *Los datos clínicos son inespecíficos y no permiten orientar un diagnóstico sindrómico.*

Presentación
Información General
Metodología
Casos Clínicos
Caso clínico 1
Caso clínico 2
Caso clínico 3
Caso clínico 4
Caso clínico 5
Algoritmos
Bibliografía de apoyo
Salir

Rinosinusitis bacteriana aguda

- Inflamación de la mucosa o del hueso subyacente de las paredes de uno o más de los senos paranasales y fosas (rinosinusitis) o simplemente la presencia de derrame en su interior.
- Se inicia en el contexto de una inflamación de la mucosa nasofaríngea, habitualmente de origen viral, que cierra los conductos de drenaje. De **0,5 a 2% de las rinitis infecciosas agudas** se complican con una rinosinusitis bacteriana:
  - Adultos: maxilar, etmoidal, frontal (desarrollo entre 4-8 años) y esfenoidal (presente a partir de 5 años).
  - Niños: etmoidal.
- El diagnóstico de rinosinusitis aguda bacteriana se basa en la **historia y examen físico**.
- No hay ningún síntoma o signo suficientemente específico para identificar el proceso como bacteriano. Es frecuente el error diagnóstico y el sobretratamiento con antibióticos.

semFYC Sociedad Española de Medicina de Familia y Comunitaria

Presentación
Información General
Metodología
Casos Clínicos
Caso clínico 1
Caso clínico 2
Caso clínico 3
Caso clínico 4
Caso clínico 5
Algorítmos
Bibliografía de apoyo
Salir

Sinusitis. Factores favorecedores

- Alteraciones en la permeabilidad de los ostium o canales de drenaje (rinitis virales, atopia/alergia, hipertrofia de cornetes, poliposis nasal, tumores, etc).
- Disfunción del transporte mucociliar (frío, sequedad ambiental, síndrome del cilio inmóvil).
- Factores tóxicos, ambientales e irritativos relacionados: tabaquismo, polución, polvo, cocaína, natación, uso inapropiado de tratamientos tópicos nasales, barotraumatismo, hiperproducción de moco.
- Enfermedades sistémicas: Diabetes mellitus, inmunosupresión, linfoma, leucemia, autoinmunes o fibrosis quística.
- Procesos dentarios (10% de las sinusitis maxilares).

Presentación
Información General
Metodología
Casos Clínicos
Caso clínico 1
Caso clínico 2
Caso clínico 3
Caso clínico 4
Caso clínico 5
Algoritmos
Bibliografía de apoyo
Salir

Caso clínico 1

Diagnóstico clínico probable de rinosinusitis aguda bacteriana

Criterios mayores

Criterios Mayores	Criterios Menores
1. Secreción nasal purulenta anterior/posterior<br>2. Tos	1. Cefalea<br>2. Dolor facial<br>3. Edema periorbitario<br>4. Fiebre<br>5. Dolor dental<br>6. Otalgia<br>7. Odinofagia<br>8. Halitosis
**Diagnóstico probable:**<br>Puntuación $\geq$ 2 criterios mayores ó 1 mayor y $\geq$ 2 menores	

Manual semFYC de enfermedades infecciosas. 2010
Rev Esp Quimioter 2008;21(1):45-59

Caso clínico 1

Sinusitis:síntomas y signos asociados

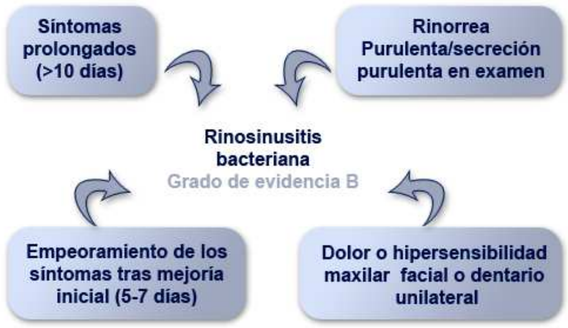

semFYC
Sociedad Española de Medicina de Familia y Comunitaria

Presentación
Información General
Metodología
Casos Clínicos
Caso clínico 1
Caso clínico 2
Caso clínico 3
Caso clínico 4
Caso clínico 5
Algoritmos
Bibliografía de apoyo
Salir

Caso clínico 1

¿Realizaría alguna prueba complementaria para establecer el diagnóstico sindrómico?

- ○ *Determinación de la proteína C reactiva (PCR)*
- ○ *Una radiología simple de senos paranasales en proyección de Waters*
- ○ *Una radiología simple de senos paranasales en proyección de Caldwell*
- ◉ *No es rentable realizar pruebas adicionales en este caso por tratarse de un cuadro no grave*
- ○ *Cultivo de moco nasal*

Caso clínico 1

Rinosinusistis bacteriana aguda. Pruebas complementarias de laboratorio

- **Reservadas para casos graves**
- **Cultivo de moco nasal**:
  - Se relaciona en menos del 30%-40% de los casos con el agente causal.
  - No se correlaciona con el cultivo de moco de los senos paranasales.
- **Cultivo del exudado por punción y aspiración del seno**:
  - Patrón oro para el diagnóstico.
  - Técnica muy invasiva, no se realiza rutinariamente.
  - Se considera significativo de infección el aislamiento de $\geq 10^4$ ufc/ml en el aspirado.

Caso clínico 1

Proteína C reactiva en la sinusitis aguda

Predicción de etiología bacteriana según punto de corte

Punto de corte	Sensibilidad	Especificidad
10 mg/L	82%	55%
25 mg/L	52%	78%

Sinusitis		
< 10 mg/L	→	viral
10 - 40 mg/L	→	duda
> 40 mg/L	→	bacteriano

Un valor de PCR < 10 mg/L en la sinusitis descarta la etiología bacteriana y 1 > 40 mg/L orienta hacia esa etiología.

Hansen JG et al. BMJ 1995;311:233-6

semFYC Sociedad Española de Medicina de Familia y Comunitaria

Presentación
Información General
Metodología
Casos Clínicos
Caso clínico 1
Caso clínico 2
Caso clínico 3
Caso clínico 4
Caso clínico 5
Algoritmos
Bibliografía de apoyo
Salir

Rinosinusistis bacteriana. Pruebas de Imagen

- **Radiología simple:**
  - Menor sensibilidad y especificidad que la clínica: sólo un 60% de las sinusitis presentan éstos signos radiológicos.
  - No permite distinguir entre etiología viral y bacteriana.
  - Diferencias interobservador muy grandes y en menores de 3 años no es útil.
  - Proyección de Waters (occipitomentoniana): visualiza mejor el seno maxilar.
  - Proyección de Caldwell (anteroposterior): visualiza mejor el seno frontal.
- **TC y RNM:** no se deben utilizar rutinariamente para el diagnostico de sinusitis, reservándose para complicaciones, procesos crónicos y diagnósticos poco claros.
- **Estarían indicadas en:**

1. Sintomatología prolongada o sin respuesta en 3-4 semanas.
2. Sinusitis clínica de repetición (> 3 veces al año).
3. Enfermedad sinusal con complicaciones orbitarias o del SNC

semFYC Sociedad Española de Medicina de Familia y Comunitaria

Presentación
Información General
Metodología
Casos Clínicos
Caso clínico 1
Caso clínico 2
Caso clínico 3
Caso clínico 4
Caso clínico 5
Algoritmos
Bibliografía de apoyo
Salir

Caso clínico 1

¿Cuál sería la etiología más probable de la infección que presenta este paciente?

- ○ *Haemophilus influenzae*
- ○ *Moraxella catarrhalis*
- ◉ *Streptococcus penumoniae*
- ○ *Mycoplasma pneumoniae*
- ○ *Rhinovirus*

Presentación
Información General
Metodología
Casos Clínicos
Caso clínico 1
Caso clínico 2
Caso clínico 3
Caso clínico 4
Caso clínico 5
Algoritmos
Bibliografía de apoyo
Salir

Sinusitis. Etiología rinosinusitis aguda

Microorganismo	Adultos	Niños
**Bacterias**		
Streptococcus penumoniae	20-43%	35-42%
Haemophilus influenzae	6-35%	21-28%
Anaerobios (dentario)	0-10%	3-7%
Staphylococcus aureus (FQ)	0-8%	-
Streptococcus pyogenes	1-3%	3-7%
Moraxella catarrhalis	2-10%	21-28%
**Virus**: *Rinovirus, Influenza, Parainfluenza*	3-15%	-
**Hongos** (IDP): *Aspergillus*	Baja	-

La sinusitis aguda es generalmente monomicrobiana, mientras que la crónica suele ser polimicrobiana. Infección mixta: 25% (combinación *S. pneumoniae* + *H. influenzae*).
La mayoría de las rinosinusitis agudas diagnosticadas en AP son de origen vírico (50%).

Caso clínico 1

¿Iniciaría tratamiento antibiótico?

- Sí
- No

semFYC Sociedad Española de Medicina de Familia y Comunitaria

Presentación
Información General
Metodología
Casos Clínicos
Caso clínico 1
Caso clínico 2
Caso clínico 3
Caso clínico 4
Caso clínico 5
Algoritmos
Bibliografía de apoyo
Salir

En relación con el antibiótico de la rinosinuistis bacteriana aguda, ¿es falso?

- *Las ventajas del tratamiento antibiótico son en general moderadas*
- *Los antibióticos logran un cambio importante en la tasa de recidivas, pero no en las recurrencias ni el paso a la cronicidad*
- *No existe evidencia que el tratamiento antibiótico inicial disminuya la frecuencia de complicaciones*
- *Los antibióticos están indicados si existe una elevada sospecha de origen dentario*
- *La antibioterapia no está indicada en las formas de sinusitis maxilar aguda leve-moderada*

Presentación
Información General
Metodología
Casos Clínicos
Caso clínico 1
Caso clínico 2
Caso clínico 3
Caso clínico 4
Caso clínico 5
Algorítmos
Bibliografía de apoyo
Salir

Antibióticos en sinusitis bacteriana

- Ventajas moderadas (NNT para la curación de 14 días).
- Los síntomas se resuelven en las dos primeras semanas en el 70-80% de lo no tratados y en 85% de los tratados.
- 15% de los pacientes presentan síntomas de rinosinusitis durante más de dos semanas, con o sin tratamiento antibiótico.
- La incidencia de complicaciones graves y la progresión a cronicidad es extremadamente baja.
- Los antibióticos no han demostrado disminuir el riesgo de complicaciones, recurrencias, recidivas o progresión a la cronicidad.

Young J. Lancet. 2008 ;371(9616):908-1
Ah-See K. Sinusitis (acute). Clin Evid (Online). 2008
Ahovuo-Saloranta A. Cochrane Database of Systematic Reviews 2009. Issue 2

Caso clínico 1

Rinosinusitis bacteriana. Indicaciones de tratamiento

- **Tratamiento antibiótico si:**
  - Clínica (≥ 7 - 10 días) con síntomas de moderados a graves (Tª >38.3°C, dolor severo) que reúnan al menos dos o más criterios de etiología bacteriana.
  - Empeoramiento clínico progresivo.
- **Seguimiento/observación en las formas aguda leve-moderada**

Rosenfeld RM. Otolaryngol Head Neck Surg. 2007 ;137(3 Suppl):S1-31
Rev Esp Quimioter. 2008;21(1):45-59

Caso clínico 1

¿Qué tratamiento considera más indicado en esta paciente?

- *Amoxicilina 1gr/8h, 7-10 días (VO)*
- *Amoxicilina-clavulánico 875mg/125 mg/8h, 7-10 días (VO)*
- *Telitromicina 800mg oral /24h, 7-10 días (VO)*
- *Claritromicina 500 mg oral /12h, 10 días (VO)*
- *Moxifloxacino 400 mg/24 h ,7-10 días (VO)*

Presentación
Información General
Metodología
Casos Clínicos
Caso clínico 1
Caso clínico 2
Caso clínico 3
Caso clínico 4
Caso clínico 5
Algoritmos
Bibliografía de apoyo
Salir

El paciente acude 3 días después a la consulta, sin experimentar mejoría del cuadro y de la afectación general. ¿Qué tratamiento le indicaría?

- ○ *Mantendría el mismo antibiótico pues es previsible una mejoría a partir del 3º día*
- ○ *Cambiaría a amoxicilina/clavulánico 500/8h, 7-10d*
- ○ *Cambiaría a levofloxacino 500 mg/24 h, 7-10 d*
- ○ *Cambiaría a cefpodoxime proxetilo 200 mg/12h 7-10d*
- ◉ *Cambiaría a amoxicilina/clavulánico 875/8h, 7-10d*

semFYC Sociedad Española de Medicina de Familia y Comunitaria

Caso clínico 1

Sinusitis. Pautas de tratamiento

Presentación	Elección	Alternativos
Aguda no complicada, sin comorbilidad, inmunocompetente y sin uso reciente de antibioterapia	Amoxicilina 875-1.000mg/8h/10d/vo	Alérgicos a penicilinas: levofloxacino (500mg/24h/10-14 d/vo) o azitromicina (500 mg/24h/3d/vo) o claritromicina (500mg/12h/10 d/ vo)
Si poca o ninguna respuesta a las 72h	Amoxicilina-ác. clavulánico (1.000-125mg/8h/10d/vo)	Similar a alérgicos
Aguda recurrente, crónica u origen dental	Amoxicilina-ác. clavulánico (1.000-125mg/8h/10d/vo)	Levofloxacino (500 mg/24h/10-14 d/vo) y si origen dental clindamicina (600mg/8h/10 d/vo)

Manual de enfermedades infecciosas en Atención Primaria, 3ª ed. Barcelona: semfyc ediciones, 2010

- **La amoxicilina es el tratamiento de elección.**
- No se han demostrado diferencias significativas entre el uso de amoxicilina frente a nuevos antibióticos, ni frente a los que teóricamente presentan ventajas con respecto a neumococo resistente a penicilinas y gérmenes productores de betalactamasas.
- La duración de la terapia antibiótica es controvertida, aunque generalmente se recomiendan 8-10 días. En pacientes con respuesta lenta es aconsejable prolongarlo hasta 2 ó 3 semanas.

Caso clínico 1

Sinusitis. Medidas generales

No existen suficientes evidencias sobre tratamientos coadyuvantes, para acortar el tiempo de duración, aunque en determinados casos pueden producir un alivio de la sintomatología:

- **Analgésicos/AINE** para alivio del dolor.
- **Vasoconstrictores** (oximetazolina) nasales, menos de 5 días.
- **Lavados nasales** con suero fisiológico para favorecer el drenaje de secreciones.
- **Antihistamínicos orales:** sólo pueden considerarse en caso de sinusitis crónica con evidente componente alérgico.
- **Corticoides sistémicos.**
- **Mucolíticos o fluidificantes:** No clara evidencia sobre su utilidad.
- Mantener una adecuada **hidratación** (6-10 vasos de líquido al día) y cierto grado de humedad en el hogar e incluso inhalación de vapor en la ducha.
- Dormir con la cabeza más elevada y evitar inhalar humo de tabaco o aire demasiado frío o seco.

Presentación
Información General
Metodología
Casos Clínicos
Caso clínico 1
Caso clínico 2
Caso clínico 3
Caso clínico 4
Caso clínico 5
Algoritmos
Bibliografía de apoyo
Salir

Sinusitis complicada

- **Criterios de derivación:**
  - Sospecha o evidencia de complicaciones orbitarias o intracraneales.
  - Fiebre alta o estado tóxico.
  - Dolor facial intenso.
  - Fallo en el tratamiento.
  - Rinosinusitis recurrente o crónica.
  - Inmunosupresión.
- **Tratamiento quirúrgico**: Aproximadamente el 5% de las sinusitis no se pueden resolver con tratamiento médico pudiéndose plantear la cirugía endoscópica con la finalidad de normalizar la función nasosinusal.

Presentación
Información General
Metodología
Casos Clínicos
Caso clínico 1
Caso clínico 2
Caso clínico 3
Caso clínico 4
Caso clínico 5
Algoritmos
Bibliografía de apoyo
Salir

Manejo de la rinosinusitis bacteriana aguda

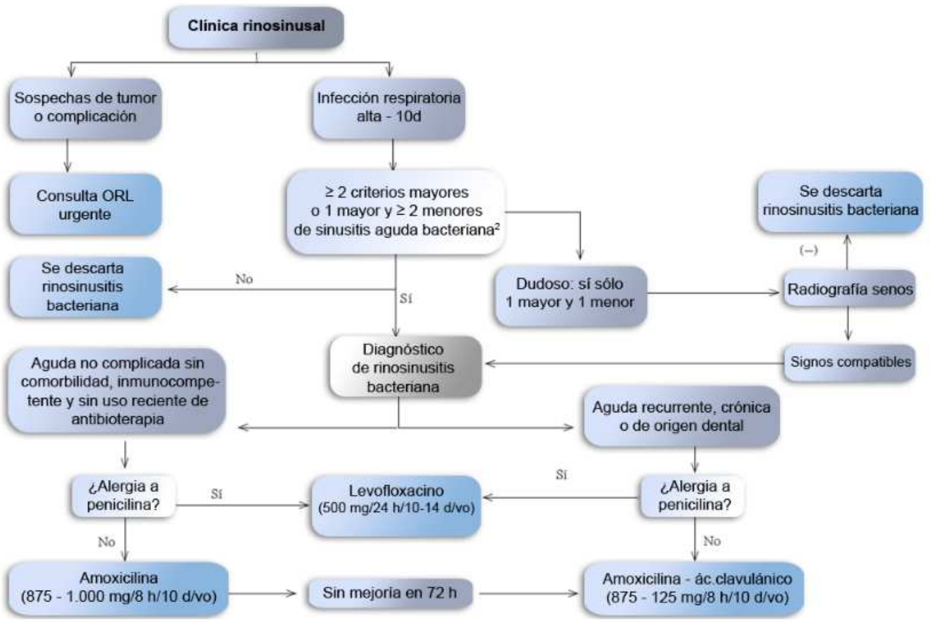

[2] *Ver diapositiva de diagnóstico clínico*

Manual de enfermedades infecciosas en Atención Primaria, 3ª ed. Barcelona: semfyc ediciones, 2010

Presentación
Información General
Metodología
Casos Clínicos
Caso clínico 1
Caso clínico 2
Caso clínico 3
Caso clínico 4
Caso clínico 5
Algoritmos
Bibliografía de apoyo
Salir

Rinosinusitis. Grados de recomendación

- El diagnóstico de rinosinusitis bacteriana debe ser reservado para los pacientes con síntomas de más de siete días, rinorrea purulenta con dolor o hipersensibilidad maxilar, especialmente si es unilateral, y empeoramiento de los síntomas después de una mejoría inicial (B).
- Los descongestionantes nasofaríngeos tópicos son útiles para reducir congestión nasal en la rinitis catarral aguda y no deben exceder los 5 días de tratamiento (B).
- El tratamiento sintomático con descongestionantes, anticolinérgicos y corticoides tópicos y los lavados nasales con soluciones hipertónicas pueden ser útiles para mejorar los síntomas de la rinosisnusitis, aunque no afectan a la duración enfermedad (C).
- La aspiración de los senos maxilares vía transnasal es el patrón oro para el diagnóstico; sin embargo, esta técnica, al ser muy invasiva, no se debe practicar de forma rutinaria (A).
- Si bien los signos clínicos y síntomas de la sinusitis aguda difícilmente discrimina entre infección viral y bacteriana, dicha evaluación sigue siendo el mejor enfoque para el diagnóstico de sinusitis aguda (A).
- El examen radiológico convencional de los senos no está recomendado de forma rutinaria para el diagnostico de sinusitis aguda debido a sus limitaciones diagnósticas y su baja probabilidad post-test (B).
- El uso de antibióticos para la sinusitis aguda confiere un pequeño beneficio terapéutico sobre el placebo con un correspondiente aumento en el riesgo de eventos adversos (A).
- Los antibióticos no logran un cambio importante en las curaciones clínicas, ni existen diferencias en el número de complicaciones, las tasas de recidivas y recurrencias en los (A).
- Los antibióticos sólo están recomendados en pacientes con una mayor probabilidad de enfermedad bacteriana que tienen síntomas persistentes (7 -10 días) o graves y reúnan al menos dos o más criterios de etiología bacteriana (A).
- Cuando está indicado, la amoxicilina durante 10-14 días, es el tratamiento de elección en función de su eficacia y eficiencia (A).
- Las fluoroquinolonas de 3ª y 4ª generación, no confieren ningún beneficio sobre beta-lactámicos, por tanto su uso como tratamiento de primera línea no está indicado (A).

Caso clínico 1

¿Cuál es el diagnóstico más probable?

- *Rinitis aguda.*
- *Bronquitis aguda*
- *Rinosinusitis aguda bacteriana.*
- *Traqueítis.*
- *Los datos clínicos son inespecíficos y no permiten orientar un diagnóstico sindrómico.*

¿Realizaría alguna prueba complementaria para establecer el diagnóstico sindrómico?

- *Determinación de la proteína C reactiva (PCR)*
- *Una radiología simple de senos paranasales en proyección de Waters*
- *Una radiología simple de senos paranasales en proyección de Caldwell*
- *No es rentable realizar pruebas adicionales en este caso por tratarse de un cuadro no grave*
- *Cultivo de moco nasal*

¿Cuál sería la etiología más probable de la infección que presenta este paciente?

- *Haemophilus influenzae*
- *Moraxella catarrhalis*
- *Streptococcus penumoniae*
- *Mycoplasma pneumoniae*
- *Rhinovirus*

¿Iniciaría tratamiento antibiótico?

- *Sí*
- *No*

En relación con el antibiótico de la rinosinuistis bacteriana aguda, ¿es falso?

- *Las ventajas del tratamiento antibiótico son en general moderadas*
- *Los antibióticos logran un cambio importante en la tasa de recidivas, pero no en las recurrencias ni el paso a la cronicidad*
- *No existe evidencia que el tratamiento antibiótico inicial disminuya la frecuencia de complicaciones*
- *Los antibióticos están indicados si existe una elevada sospecha de origen dentario*
- *La antibioterapia no está indicada en las formas de sinusitis maxilar aguda leve-moderada*

¿Qué tratamiento considera más indicado en esta paciente?

- *Amoxicilina 1gr/8h, 7-10 días (VO)*
- *Amoxicilina-clavulánico 875mg/125 mg/8h, 7-10 días (VO)*
- *Telitromicina 800mg oral /24h, 7-10 días (VO)*
- *Claritromicina 500 mg oral /12h, 10 días (VO)*
- *Moxifloxacino 400 mg/24 h ,7-10 días (VO)*

El paciente acude 3 días después a la consulta, sin experimentar mejoría del cuadro y de la afectación general. ¿Qué tratamiento le indicaría?

- *Mantendría el mismo antibiótico pues es previsible una mejoría a partir del 3º día*
- *Cambiaría a amoxicilina/clavulánico 500/8h, 7-10d*
- *Cambiaría a levofloxacino 500 mg/24 h, 7-10 d*
- [illegible]

¿Quiere modificar o repasar las preguntas que ha respondido durante el Caso Clínico?
Estas preguntas finales, tendrán carácter evaluatorio

Sí No

Presentación
Información General
Metodología
Casos Clínicos
Caso clínico 1
Caso clínico 2
Caso clínico 3
Caso clínico 4
Caso clínico 5
Algoritmos
Bibliografía de apoyo
Salir

Caso clínico superado

Informe de respuestas a las preguntas test

Pregunta	Respuesta Previa	Respuesta Definitiva	Respuesta Verdadera
¿Cuál es el diagnóstico más probable?	✓	✓	
¿Realizaría alguna prueba complementaria para establecer el diagnóstico sindrómico?	✓	✓	
¿Cuál sería la etiología más probable de la infección que presenta este paciente?	✓	✓	
¿Iniciaría tratamiento antibiótico?	✓	✓	
En relación con el antibiótico de la rinosinuistis bacteriana aguda, ¿es falso?	✓	✓	
¿Qué tratamiento considera más indicado en esta paciente?	✓	✓	
El paciente acude 3 días después a la consulta, sin experimentar mejoría del cuadro y de la afectación general. ¿Qué tratamiento le indicaría?	✓	✓	
	7 de 7	7 de 7	

Tiempo total usado:

01:31:06
