# Supplementary material for: Impact of a multifaceted intervention to improve antibiotic prescribing: a pragmatic cluster-randomised controlled trial
Source: Antimicrob Resist Infect Control. 2020 Dec 7;9:195. doi: 10.1186/s13756-020-00857-9 (PMC7722452; doi:10.1186/s13756-020-00857-9)
Supplement: Supplementary file 3 — Additional file 3. Supplementary intervention. [file 13756_2020_857_MOESM3_ESM.pdf]

Appendix 3 – Supplementary Intervention

Supplementary Figure 1. Timeline of each of the activities included in the multifaceted intervention.

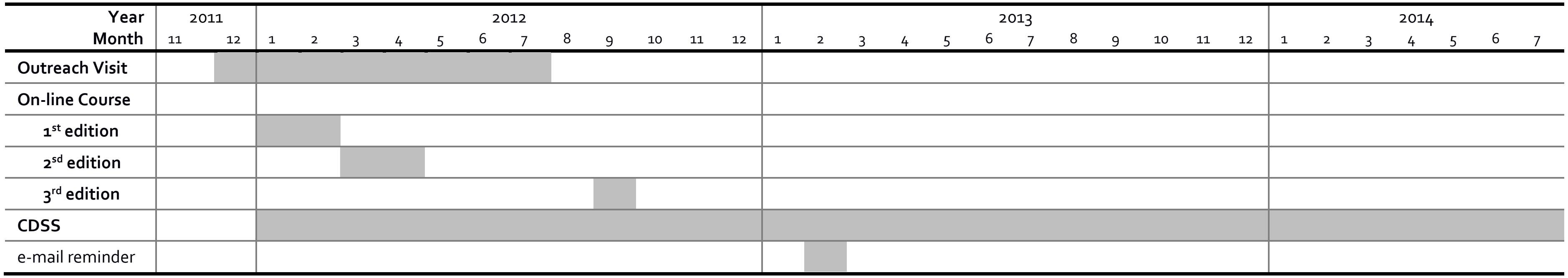

Abbreviations. CDSS; Clinical decision support system.
