# Supplementary material for: Impact of a multifaceted intervention to improve antibiotic prescribing: a pragmatic cluster-randomised controlled trial
Source: Antimicrob Resist Infect Control. 2020 Dec 7;9:195. doi: 10.1186/s13756-020-00857-9 (PMC7722452; doi:10.1186/s13756-020-00857-9)
Supplement: Supplementary file 4 — Additional file 4. Presentation of outreach visit. [file 13756_2020_857_MOESM4_ESM.pdf]

MINISTERIO DE CIENCIA E INNOVACIÓN
Instituto de Salud Carlos III
FONDO EUROPEO DE DESARROLLO REGIONAL
"Una manera de hacer Europa"

Rational use of antibiotics in primary care

CRISTIAN GONZÁLEZ GONZÁLEZ
Departamento de Medicina Preventiva y Salud Pública
Universidade de Santiago De Compostela

Background

Importance

2.3.2 Human burden of antibiotic resistance

The estimated human burden of infections due to the selected antibiotic-resistant bacteria is presented in Table 2.

The study confirmed that MRSA was, in 2007, the most common, single, multidrug-resistant bacterium in the EU as per the estimated number of cases of infection due to this bacterium. However, the sum of cases of antibiotic-resistant Gram-positive bacteria (mostly MRSA and vancomycin-resistant *Enterococcus faecium*) was comparable to that of antibiotic-resistant Gram-negative bacteria (third-generation cephalosporin-resistant *E. coli* and *K. pneumoniae*, and carbapenem-resistant *P. aeruginosa*).

Overall, it was estimated that in 2007 approximately 25 000 patients died from an infection due to any of the selected frequent antibiotic-resistant bacteria in the EU, Iceland and Norway. Notably, about two thirds of these deaths were caused by infections due to Gram-negative bacteria. In addition, infections due to any of the selected antibiotic-resistant bacteria resulted in approximately 2.5 million extra hospital days.

2.3.3 Economic burden of antibiotic resistance

The estimated economic burden of infections due to the selected antibiotic-resistant bacteria is presented in Table 3.

Based on the number of extra hospital days, extra in-hospital costs in 2007 were estimated at more than EUR 900 million in the EU, Iceland and Norway.

Based on 2007 data, outpatient care costs were estimated at about EUR 10 million and productivity losses due to absence from work of infected patients were estimated at more than EUR 150 million, each year. Productivity losses due to patients who died from their infection were estimated at about EUR 450 million each year. Overall, societal costs of infections due to the selected antibiotic-resistant bacteria were estimated at about EUR 1.5 billion each year.

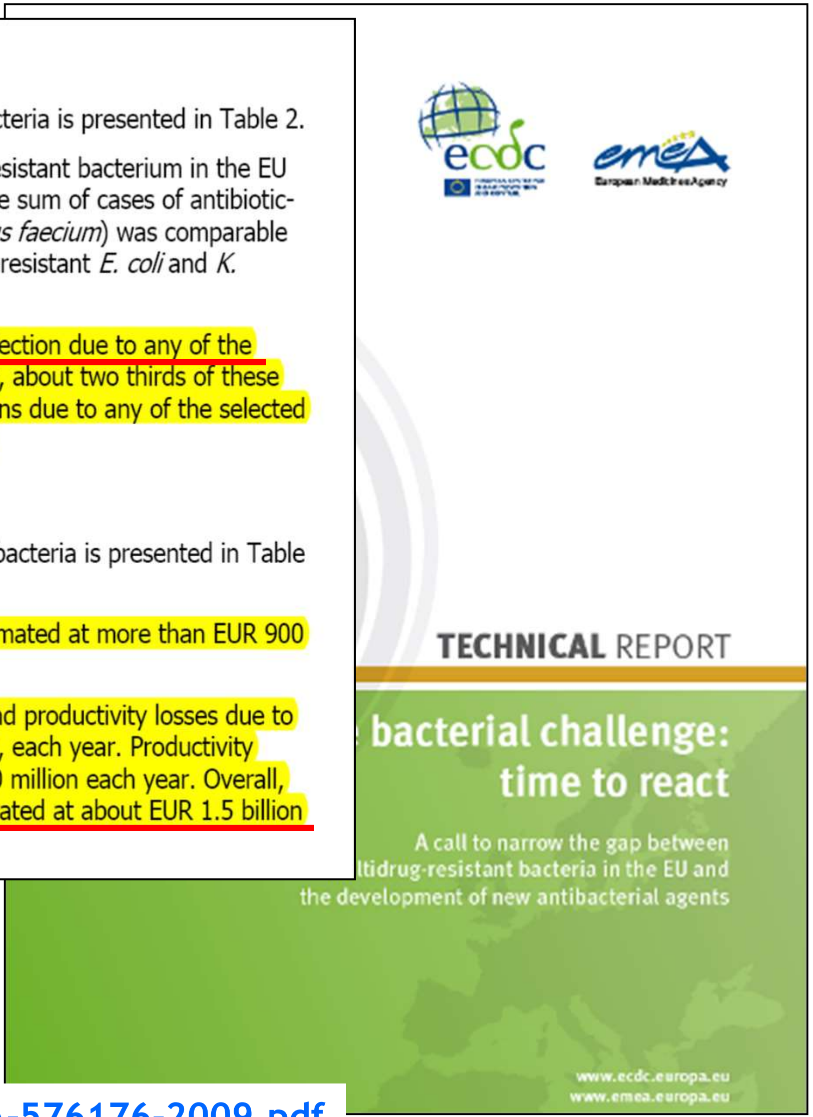

www.ema.europa.eu/pdfs/human/antimicrobial.../EMEA-576176-2009.pdf

Importance

Figure 1: 14 classes of antibiotics were introduced for human use between 1935 and 1968; since then, 5 have been introduced.

Figure 2: The number of new systemic antibiotic agents has declined since 1980, and most (75%) of these drugs are in two classes, beta-lactams and quinolones.

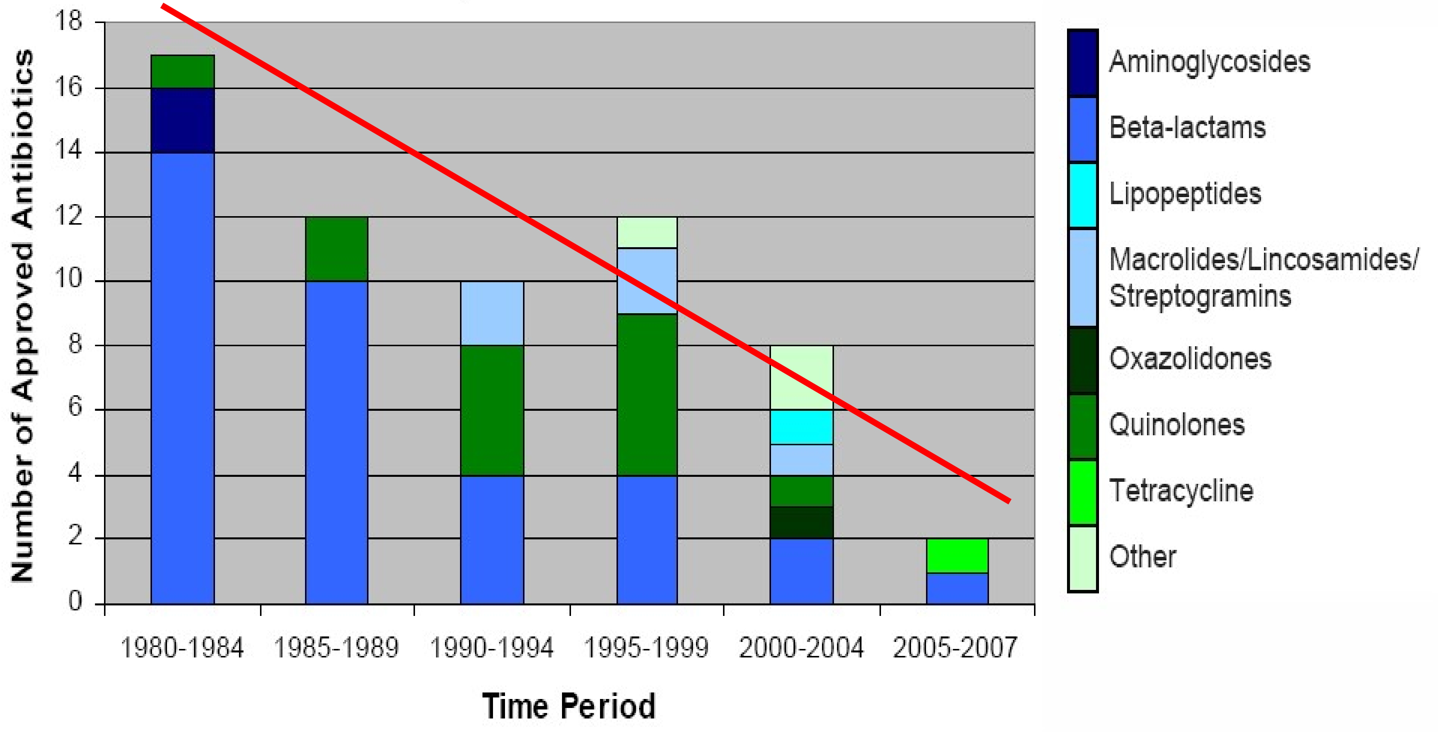

Antecedentes

Importancia

Perspectivas políticas de la OMS sobre medicamentos

10

La contención de la resistencia a los antimi

Abril de 2005
Organización Mundial de la Salud
Ginebra

El problema de la resistencia a los antimicrobianos

La resistencia a los antimicrobianos (RAM) es uno los problemas de salud pública más graves del mun Muchos de los microbios (bacterias, virus, protozoos) causan enfermedades infecciosas han dejado de resp der a los antimicrobianos de uso común (antibacterian como los antibióticos, antivíricos y antiprotozoarios). El blema es tan grave que, si no se emprende una acc concertada a escala mundial, corremos el riesgo de gresar a la era preantibiótica, época en que morían chos más niños que ahora por causa de enfermeda infecciosas y no era posible practicar intervencio quirúrgicas mayores debido al riesgo de infección. enfermedades infecciosas más importantes matan a

El problema de la resistencia a los antimicrobianos

La resistencia a los antimicrobianos (RAM) es uno de los problemas de salud pública más graves del mundo. Muchos de los microbios (bacterias, virus, protozoos) que causan enfermedades infecciosas han dejado de responder a los antimicrobianos de uso común (antibacterianos, como los antibióticos, antivíricos y antiprotozoarios). El problema es tan grave que, si no se emprende una acción concertada a escala mundial, corremos el riesgo de regresar a la era preantibiótica, época en que morían muchos más niños que ahora por causa de enfermedades infecciosas y no era posible practicar intervenciones quirúrgicas mayores debido al riesgo de infección. Las enfermedades infecciosas más importantes matan a más

Background

THE LANCET

Outpatient antibiotic use in Europe and association with resistance: a cross-national database study

Herman Goossens, Matus Ferech, Robert Vander Stichele, Monique Elseviers, for the ESAC Project Grou

Summary

Background Resistance to antibiotics is a major public-health problem and an recognised as the main selective pressure driving this resistance. Our aim was to and the association with resistance.

Methods We investigated outpatient antibiotic use in 26 countries in Eurc comparable distribution or reimbursement data, between Jan 1, 1997, and Dec 3 of defined daily doses (DDD) per 1000 inhabitants per day, according to WH classification and DDD measurement methodology. We assessed the ecological and antibiotic resistance rates using Spearman's correlation coefficients.

Findings Prescription of antibiotics in primary care in Europe varied greatly; (32·2 DDD per 1000 inhabitants daily) and the lowest was in the Netherlands (10· We noted a shift from the old narrow-spectrum antibiotics to the new broad-spec striking seasonal fluctuations with heightened winter peaks in countries with showed higher rates of antibiotic resistance in high consuming countries, consumption in southern and eastern Europe than in northern Europe.

Interpretation These data might provide a useful method for assessing public-h antibiotic use and resistance levels.

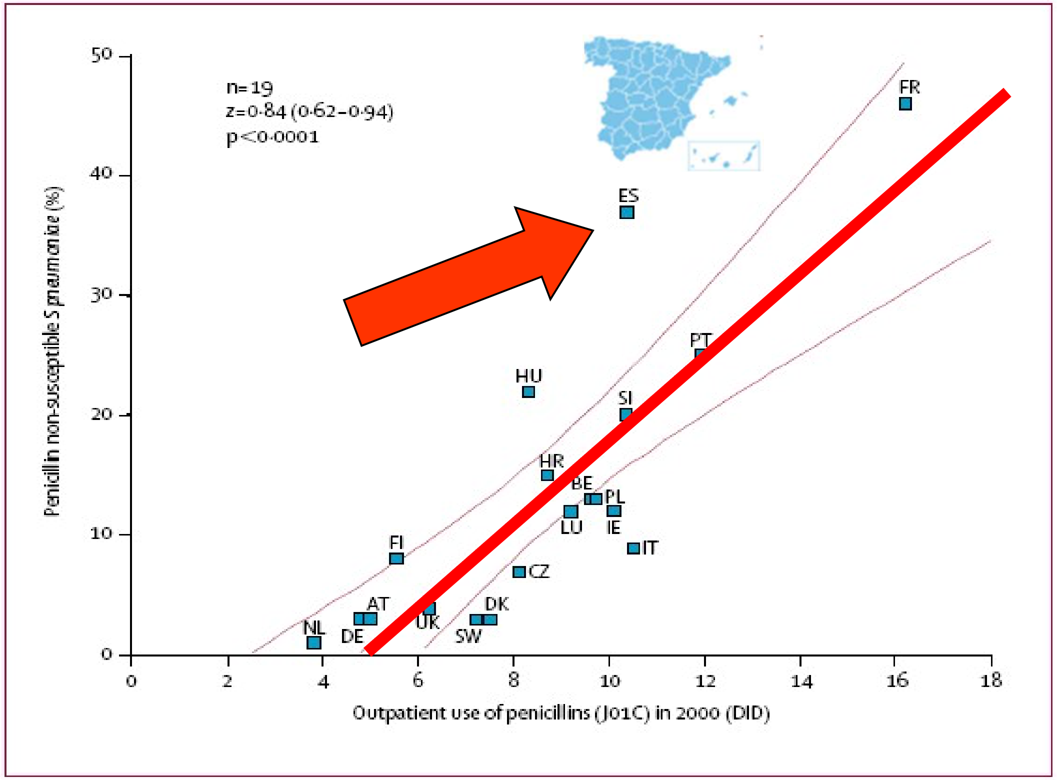

Figure 6: Correlation between penicillin use and prevalence of penicillin non-susceptible *S pneumoniae*
AT, Austria; BE, Belgium; HR, Croatia; CZ, Czech Republic; DK, Denmark; FI, Finland; FR, France; DE, Germany; HU, Hungary; IE, Ireland; IT, Italy; LU, Luxembourg; NL, The Netherlands; PL, Poland; PT, Portugal; SI, Slovenia; ES, Spain; UK, England only.

Relation of long-term consumption and resistance: co-selection of resistance.

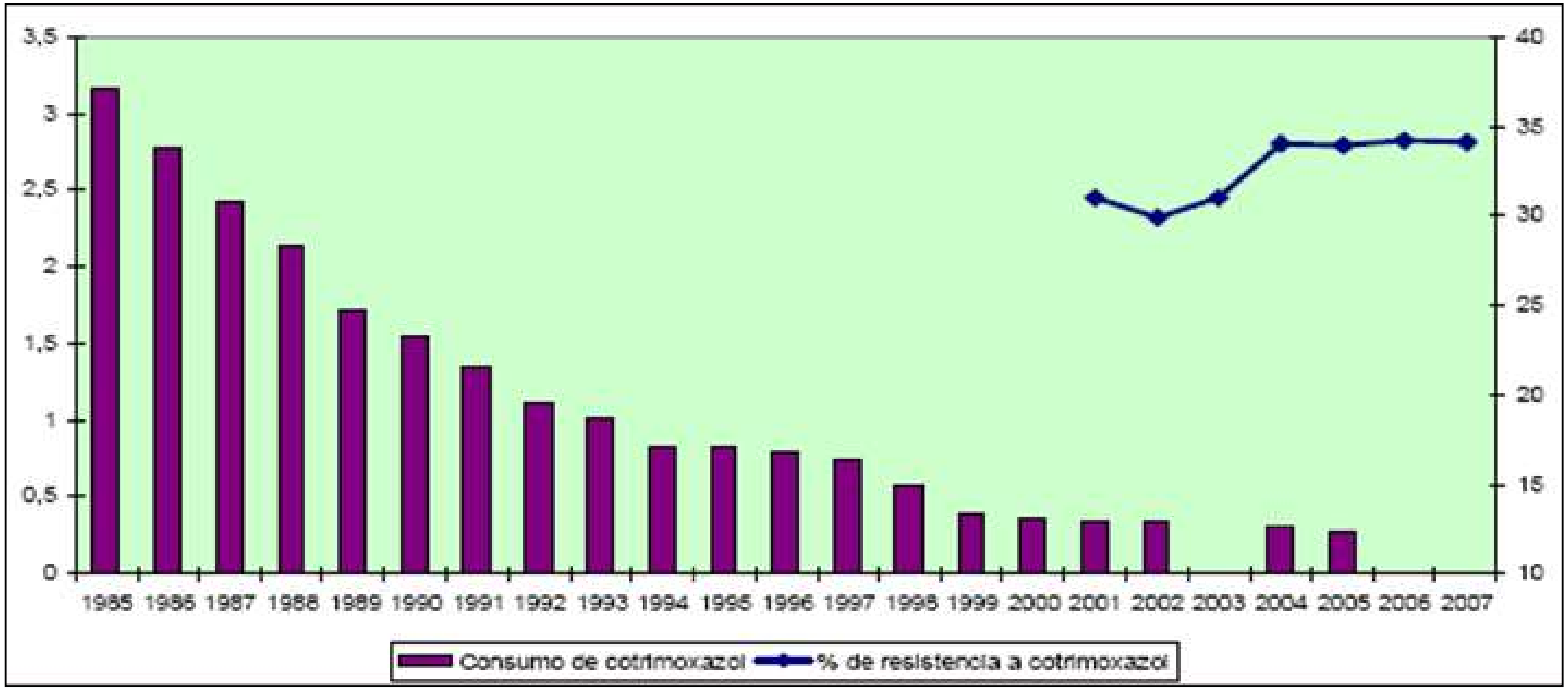

Oteo J. 2008

Background

Importance | Consumption-resistance | Europe-Spain

THE LANCET

Articles

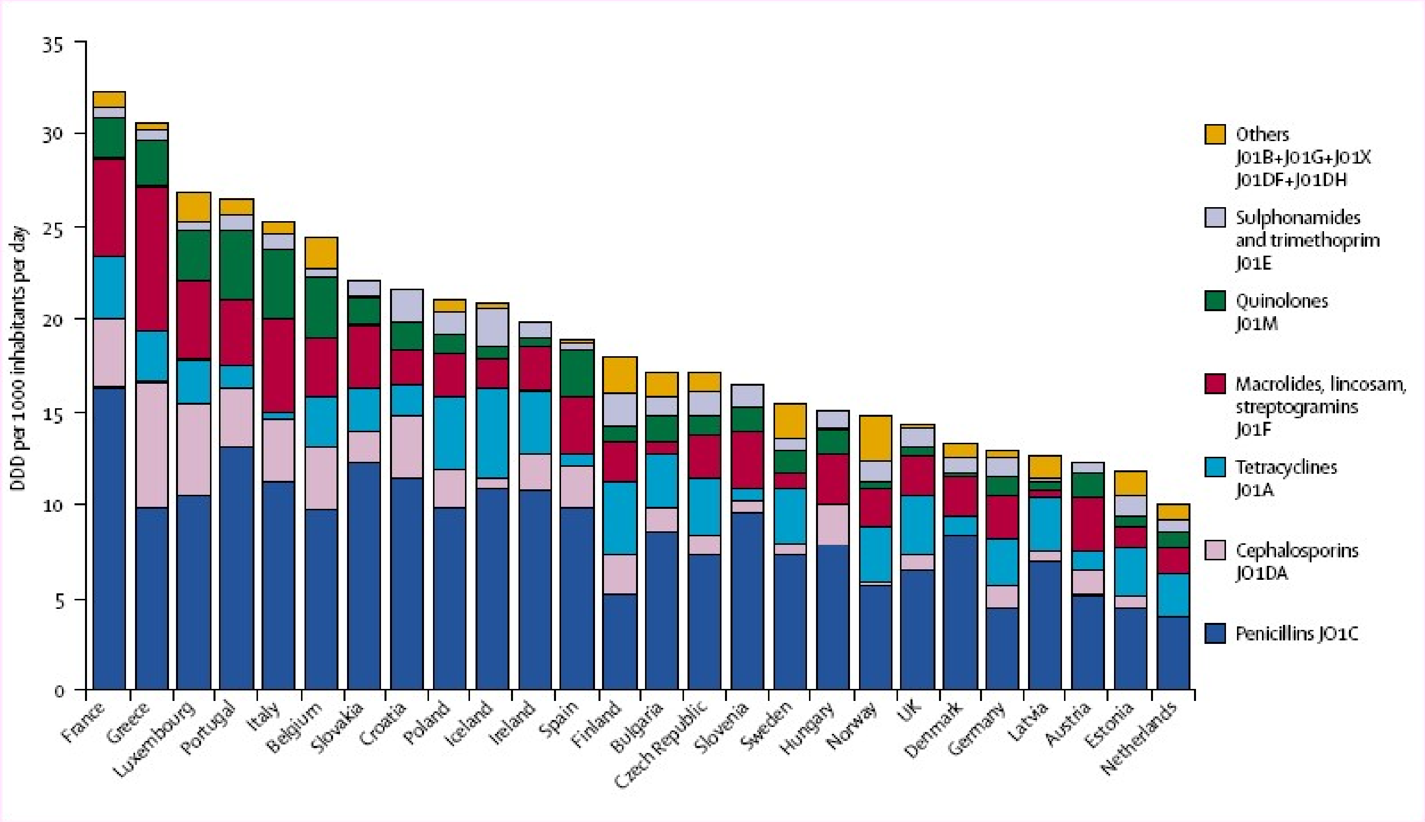

Figure 1: Total outpatient antibiotic use in 26 European countries in 2002

Lancet 2005; 365: 579–87

See Comment page 548

*Members listed at end of report

ESAC Management Team, Department of Microbiology, University of Antwerp, B-2610 Antwerp, Belgium (Prof H Goossens MD, M Ferech PharmD, R Vander Stichele MD, M Elseviers PhD); and Department of Medical Microbiology, Leiden University Medical Centre, Netherlands (H Goossens)

Correspondence to: Prof H Goossens Herman.Goossens@uza.be

Current Situation

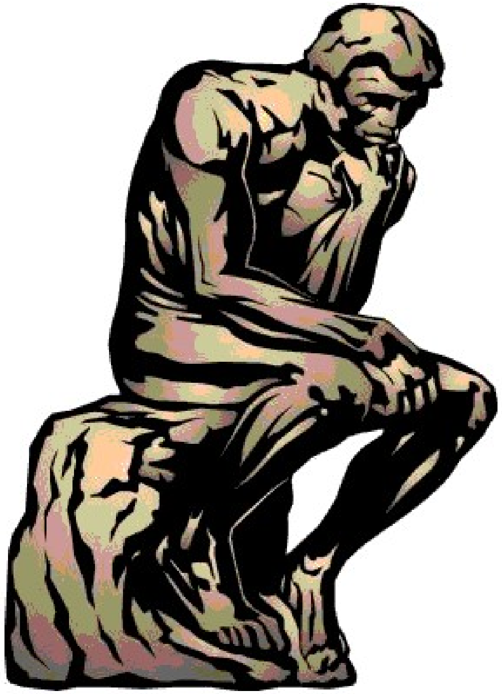

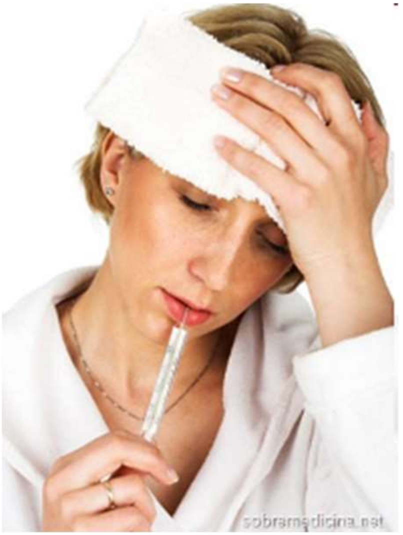

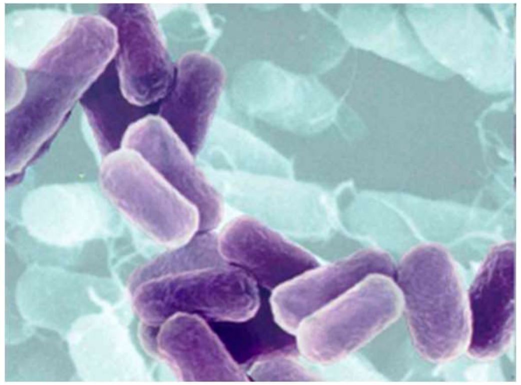

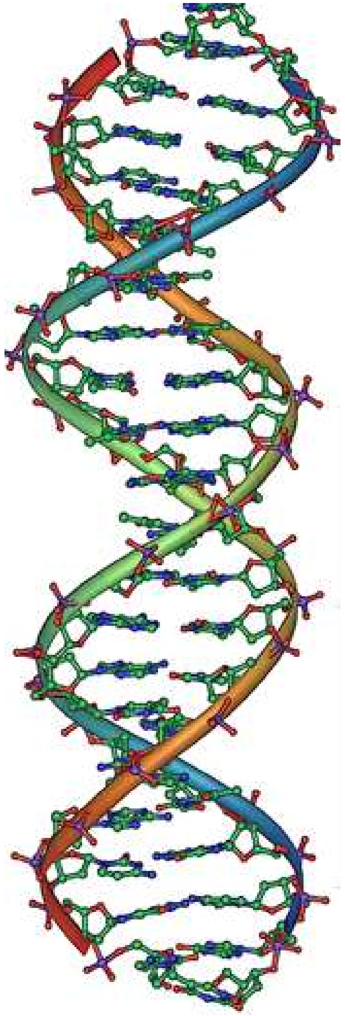

Background

Importance | Consumption-resistance | Europe-Spain

THE LANCET

Articles

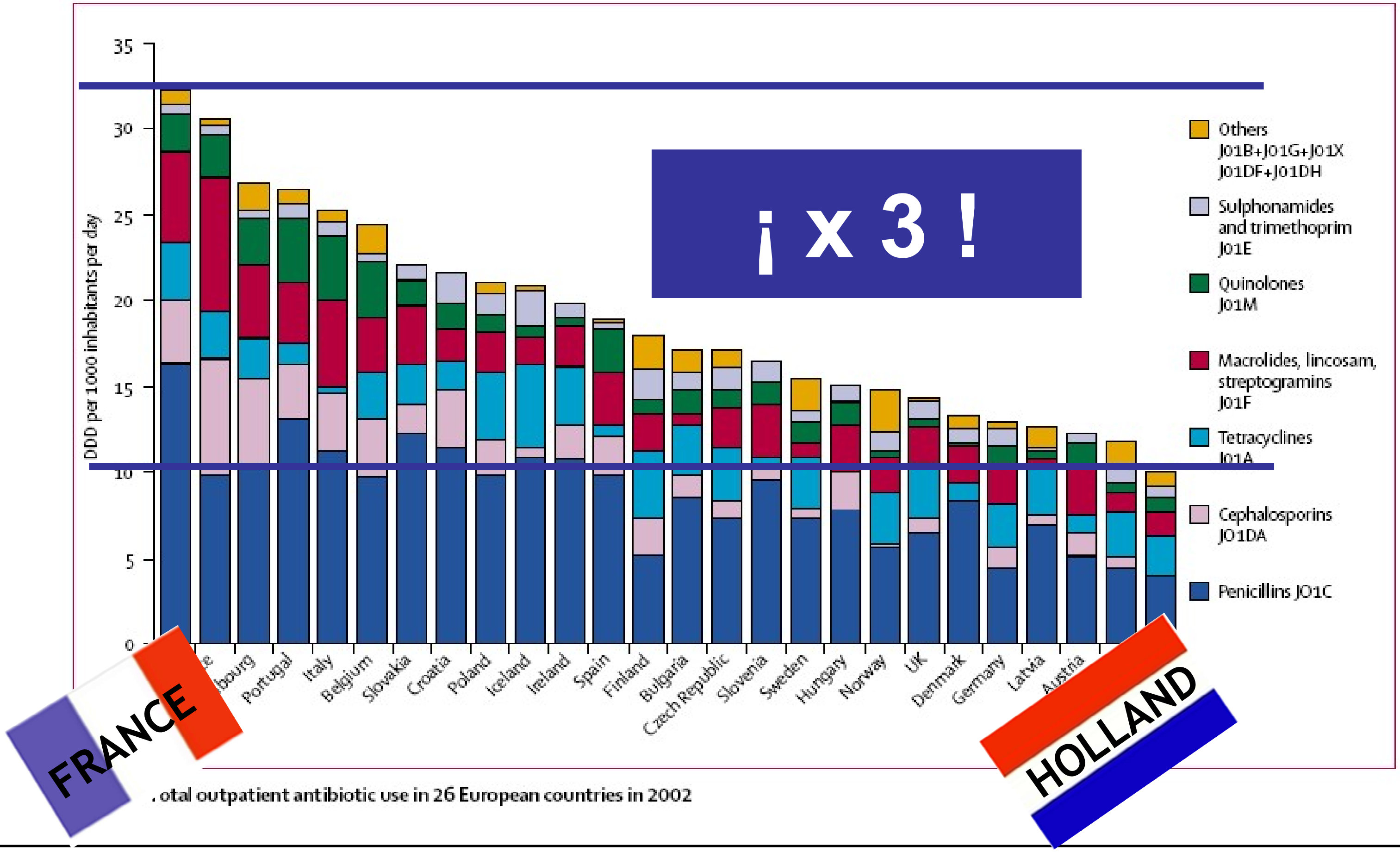

. otal outpatient antibiotic use in 26 European countries in 2002

Lancet 2005; 365: 579–87

See Comment page 548

*Members listed at end of report

ESAC Management Team, Department of Microbiology, University of Antwerp, B-2610 Antwerp, Belgium (Prof H Goossens MD, M Ferech PharmD, R Vander Stichele MD, M Elseviers PhD); and Department of Medical Microbiology, Leiden University Medical Centre, Netherlands (H Goossens)

Correspondence to: Prof H Goossens Herman.Goossens@uza.be

Current Situation

Importance | Consumption-resistance | Europe-Spain

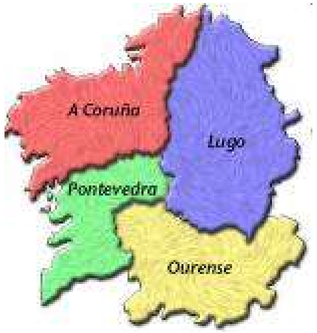

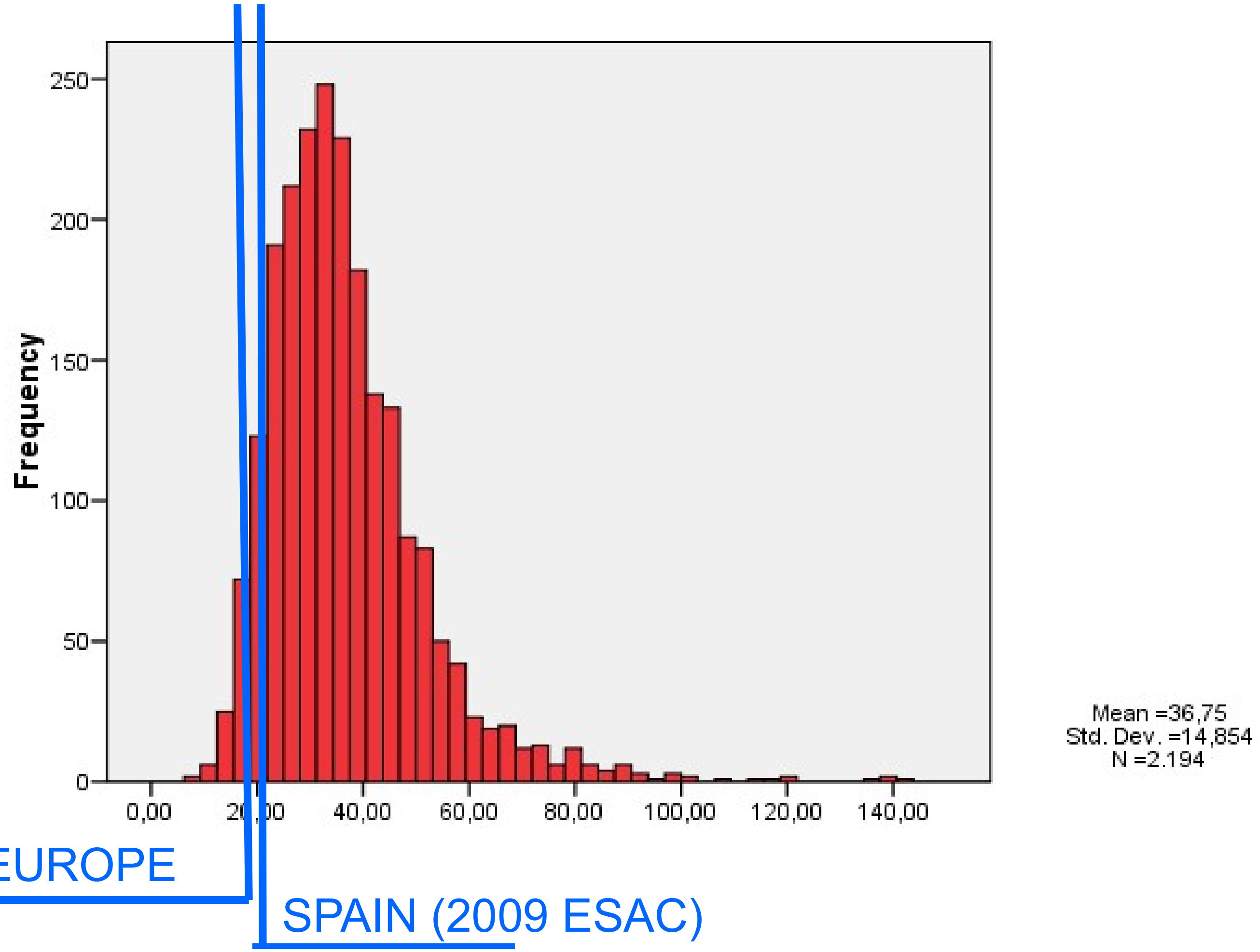

BMJ

RESEARCH

Variation in antibiotic prescribing and its impact on recovery in patients with acute cough in primary care: prospective study in 13 countries

C C Butler, professor,[1] K Hood, director,[2] T Verheij, professor,[3] P Little, professor,[4] H Melbye, professor,[5] J Nuttall, senior trial manager,[2] M J Kelly, statistician,[2] S Mölstad, professor,[6] M Godycki-Cwirko, physician,[7] J Almirall, professor,[8] A Torres, professor,[9] D Gillespie, trainee statistician,[2] U Rautakorpi, senior medical officer,[10] S Coenen, postdoctoral fellow,[11,12] H Goossens, professor[13]

[1]Department of Primary Care and Public Health, School of Medicine, Cardiff University, Cardiff CF14 4XN, Wales

[2]South East Wales Trials Unit (SEWTU), Department of Primary Care and Public Health, School of Medicine, Cardiff University, Heath Park, Cardiff, Wales

[3]University Medical Centre Utrecht, Julius Center for Health, Sciences and Primary Care, Universiteitsweg 100, Stratenum

ABSTRACT

Objective To describe variation in antibiotic prescribing for acute cough in contrasting European settings and the impact on recovery.

Design Cross sectional observational study with clinicians from 14 primary care research networks in 13 European countries who recorded symptoms on presentation and management. Patients followed up for 28 days with patient diaries.

Setting Primary care.

antibiotic prescribing is not associated with clinically important differences in recovery.

Trial registration Clinicaltrials.gov NCT00353951.

INTRODUCTION

Antibiotic resistance is a growing problem worldwide, with 10% of *Streptococcus pneumoniae* isolates recorded as non-susceptible to penicillin in 30 countries in 2007.[1] There is wide variation in antibiotic prescribing for ambulant patients in Europe.[2] We do not know if

Factors influencing the prescription of antibiotics by primary care doctors

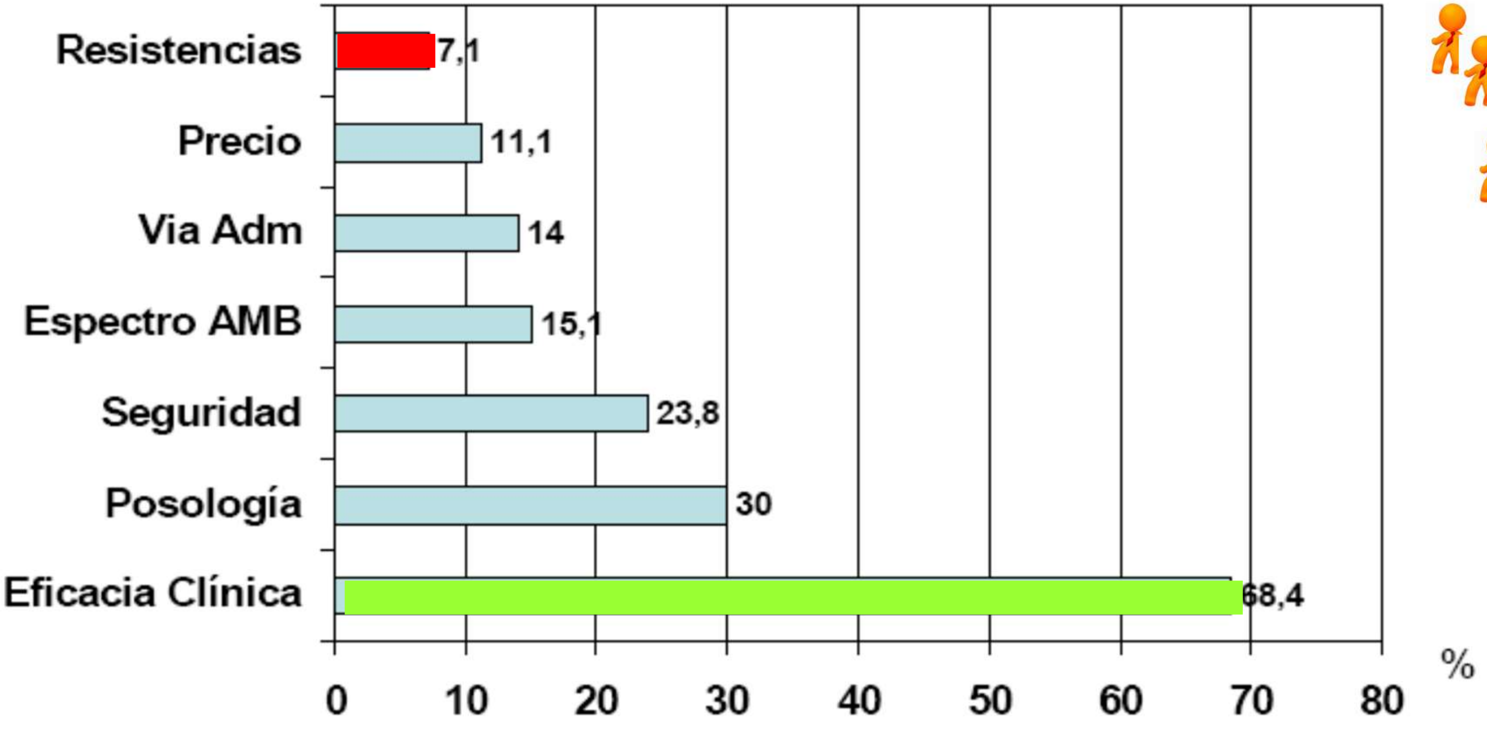

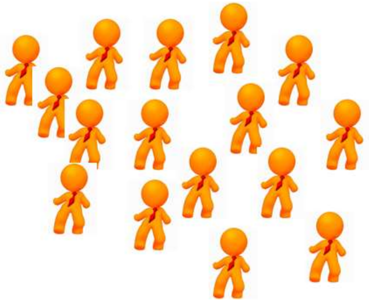

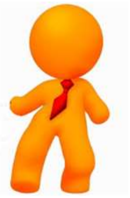

Ripoll MA et al, Rev Esp Quimioter 2008;21(1):26-31

Current Situation

Importance | Consumption-resistance | Europe-Spain | **Primary care**

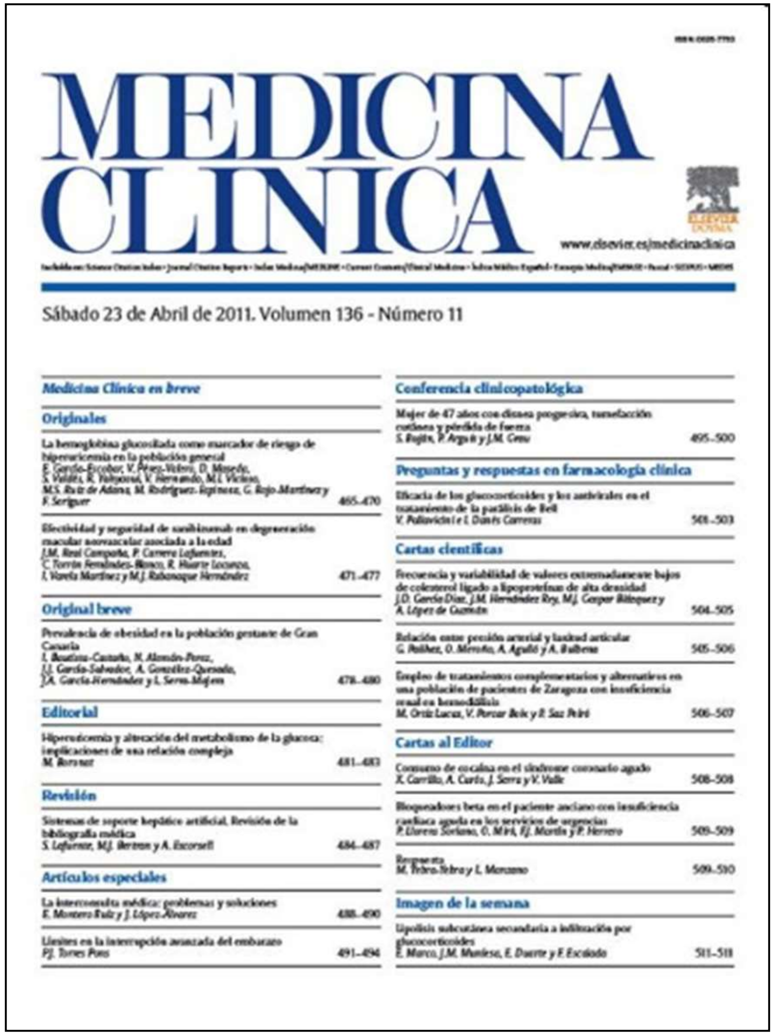

MEDICINA CLINICA

www.elsevier.es/medicinaclinica

Sábado 23 de Abril de 2011. Volumen 136 - Número 11

Medicina Clínica en breve

Originales

La hemoglobina glucosilada como marcador de riesgo de hiperuricemia en la población general
E. García-Escobar, V. Pérez-Valero, D. Mancha, S. Valdés, R. Valhurest, V. Hernando, M.L. Viclens, M.S. Ruiz de Adana, M. Rodríguez-Espinosa, G. Rojo-Martínez y F. Soriguer 465-470

Efectividad y seguridad de ranibizumab en degeneración macular neovascular asociada a la edad
J.M. Real Campaña, P. Carrera Lafuentes, C. Torrón Fernández-Blanco, R. Huarte Locanza, I. Varela Martínez y M.J. Rabanaque Hernández 471-477

Original breve

Prevalencia de obesidad en la población gestante de Gran Canaria
I. Bautista-Castaño, N. Alemán-Pérez, J.J. García-Salvador, A. González-Quesada, J.A. García-Hernández y L. Serra-Majem 478-480

Editorial

Hiperuricemia y alteración del metabolismo de la glucosa: implicaciones de una relación compleja
M. Borrás 481-483

Revisión

Sistemas de soporte hepático artificial. Revisión de la bibliografía médica
S. Lafuente, M.J. Bertran y A. Escorsell 484-487

Artículos especiales

La interconsulta médica: problemas y soluciones
E. Montero Ruiz y J. López Álvarez 488-490

Límites en la interrupción avanzada del embarazo
P.J. Torres Pons 491-494

Conferencia clinicopatológica

Mujer de 47 años con disnea progresiva, tumefacción cutánea y pérdida de fuerza
S. Bujan, P. Arguis y J.M. Grau 495-500

Preguntas y respuestas en farmacología clínica

Eficacia de los glucocorticoides y los antivirales en el tratamiento de la parálisis de Bell
V. Pallavicini e I. Danés Carreras 501-503

Cartas científicas

Frecuencia y variabilidad de valores extremadamente bajos de colesterol ligado a lipoproteínas de alta densidad
J.D. García Díaz, J.M. Hernández Rey, M.J. Casper Bilequer y A. López de Guzmán 504-505

Relación entre presión arterial y laxitud articular
G. Pelíñez, O. Mirrallo, A. Agulló y A. Balbena 505-506

Empleo de tratamientos complementarios y alternativos en una población de pacientes de Zaragoza con insuficiencia renal en hemodiálisis
M. Ortiz Lucas, V. Porcar Bele y P. Saz Peiró 506-507

Cartas al Editor

Consumo de cocaína en el síndrome coronario agudo
X. Carrillo, A. Curós, J. Serra y V. Valle 508-508

Bloqueadores beta en el paciente anciano con insuficiencia cardiaca aguda en los servicios de urgencias
P. Llorens Soriano, O. Miró, F.J. Martín y P. Herrero 509-509

Respuesta
M. Tribo-Tebra y L. Manzano 509-510

Imagen de la semana

Lipólisis subcutánea secundaria a infiltración por glucocorticoides
E. Marco, J.M. Muniesa, E. Duarte y F. Escalada 511-511

Data distribution according to percentage of correct and incorrect prescription of antibiotic in Primary Care. *Med Clin.*

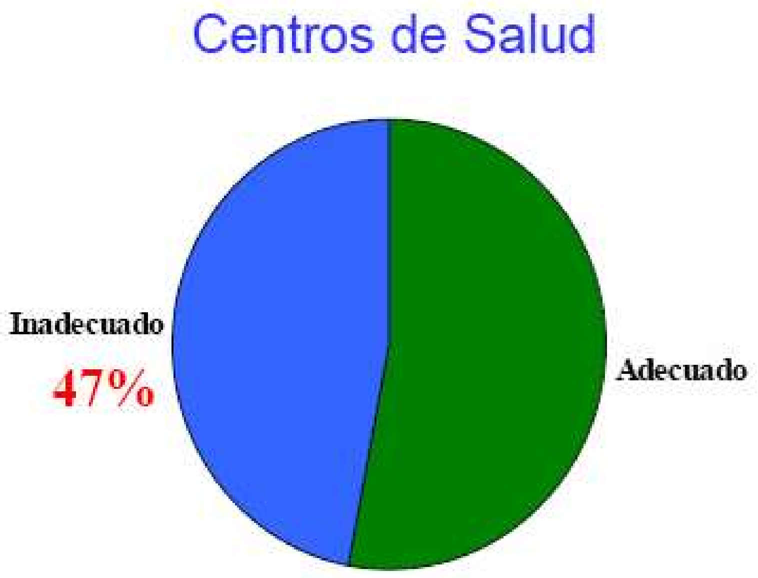

Saturno PJ. Med Clin (Barc)

CONDITIONING FACTORS OF THE PRESCRIPTION OF ANTIBIOTICS IN Primary CARE:

Patient pressure

Industrial pressure

Fear / Uncertainty

Adherence to clinical guidelines

Access to clinical tests

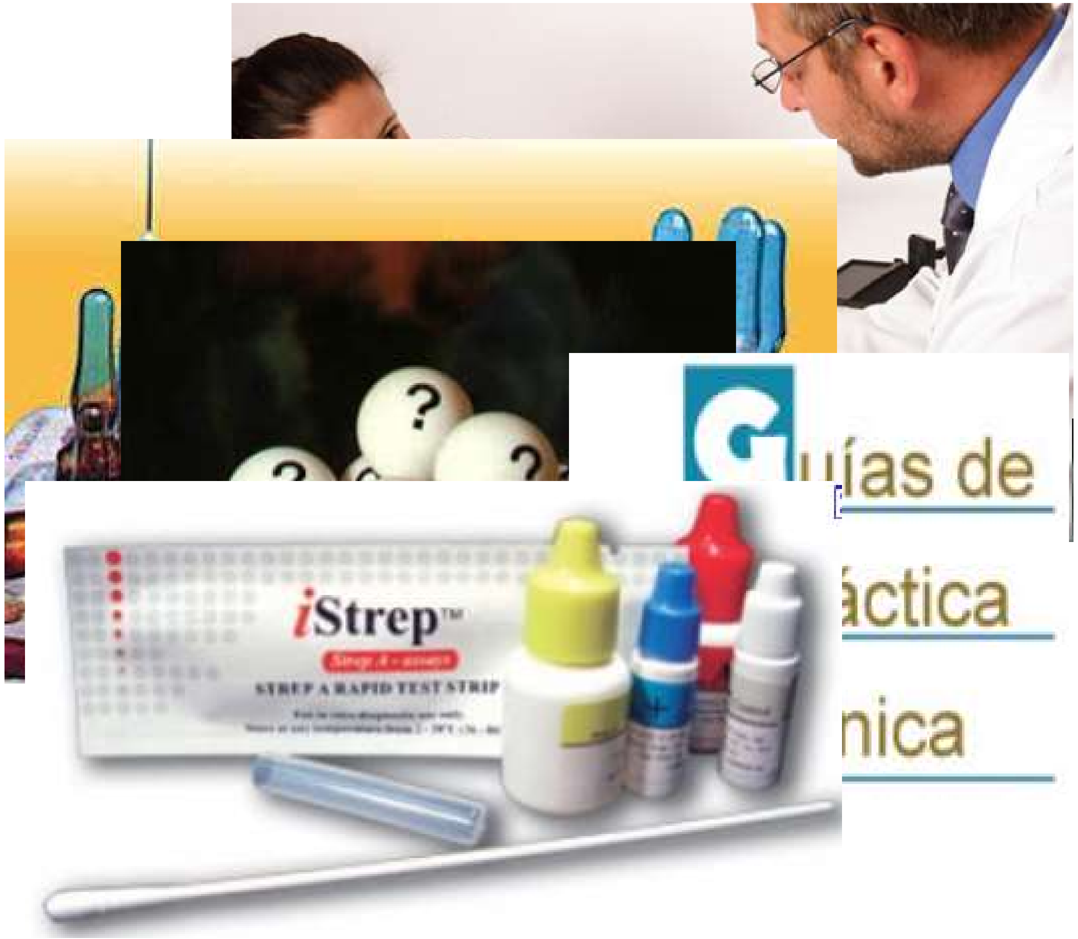

CONDITIONING FACTORS OF THE PRESCRIPTION OF ANTIBIOTICS IN Primary CARE:

Patient pressure

~~Industrial pressure~~

Fear / Uncertainty

Adherence to clinical guidelines

~~Access to clinical tests~~

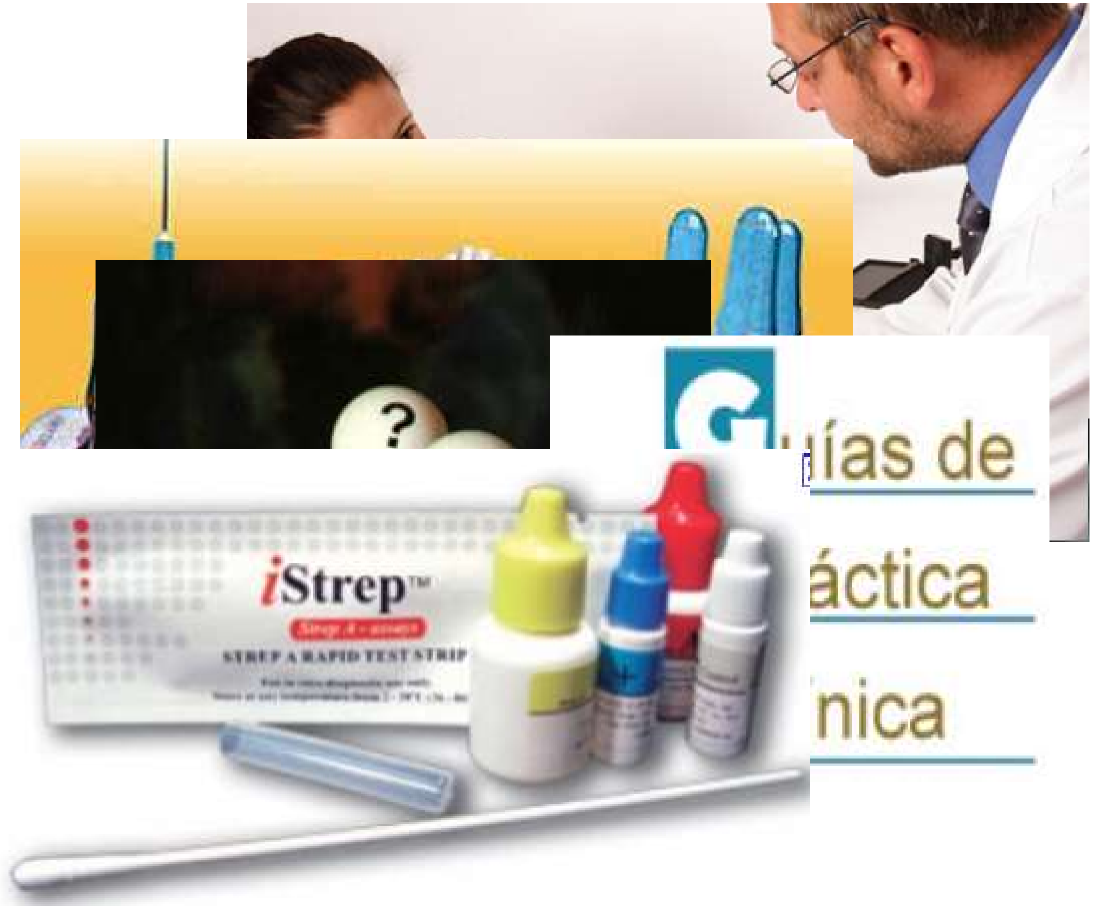

Current Situation

Importance | Consumption-resistance | Europe-Spain | Primary Care | SOLUTION

Patient pressure	Communication Skills 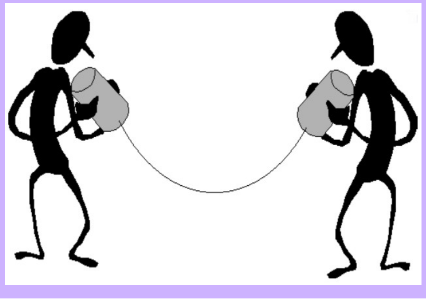
Fear / Uncertainty	
Adherance to clinical guidelines	

PATIENT PRESSURE: COMMUNICATION SKILLS

Improve patient-doctor communication.

In 50% of the cases:

.- NO detected complaints

.- Patient and doctor. NO agreement

The patient can not describe his/her problem

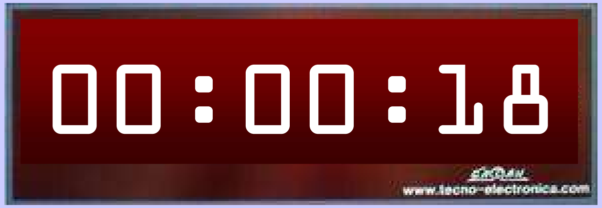

BMJ

he Toronto consensus

vart, Peter Maguire, Mack Lipkin, Dennis Novack,

substantial deficiencies when studied. Only a low proportion of visits with doctors include any patient education,[15] and a surprisingly high proportion of patients do not understand or remember what their physicians tell them about diagnosis and treatment.[16] Cultural differences also impede the work with patients.[17 18]

Patient anxiety and dissatisfaction is related to uncertainty and lack of information, explanation, and feedback from the doctor. Yet doctors often misperceive the amount and type of information patients want. The language doctors use is often unclear, both as regards the use of jargon and in relation to a lack of the expected shared meanings of relatively common terms.[20-23]

Cancer Research
Campaign Psychological

practice.

This consensus statement addresses three issues:

PATIENT PRESSURE: COMMUNICATION SKILLS

✓Non-verbal behavior:

- Impersonal tone of voice
- Rude and distant
- Speak rapidly
- G [illegible]ipe book))
- P [illegible]

For this, antibiotic is not needed

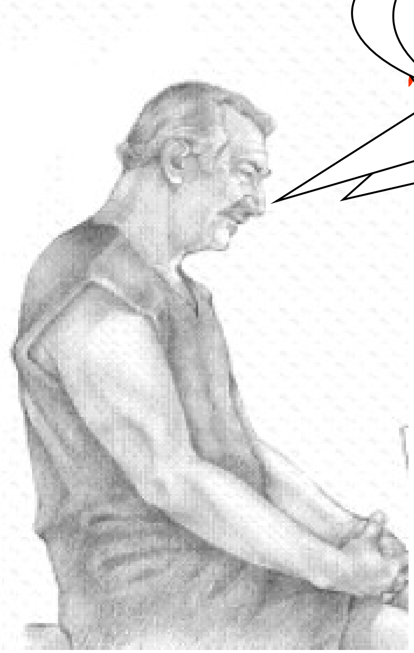

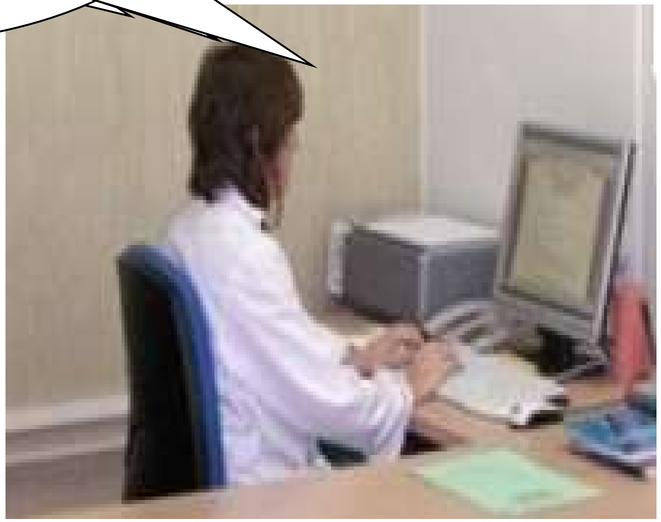

Little ASSERTIVITY

Low EMPATHY

No Point of AGREEMENT

Outcome:

***Poor adherence** to treatment*

Consequences for the patient:

***Passivity** towards doctor's recommendations*

Consequences for the physician:

***NO Closeness** to the patient*

Burn out professional

PATIENT PRESSURE: COMMUNICATION SKILLS

✓Non-verbal behaviour:

Conversational voice tone

Speak fluently

First person messa[illegible]

Patient-c[illegible]

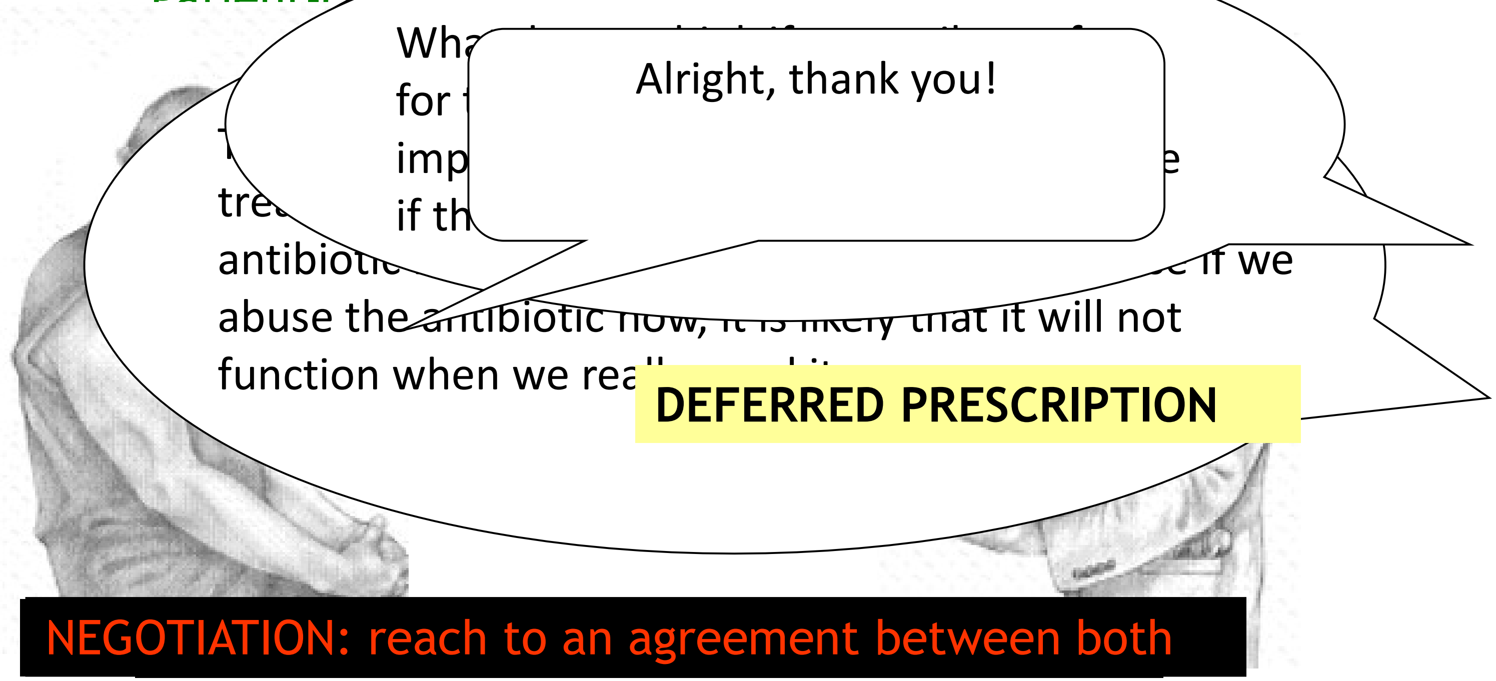

NEGOTIATION: reach to an agreement between both

ASSERTIVENESS

EMPATHY

NEGOTIATION

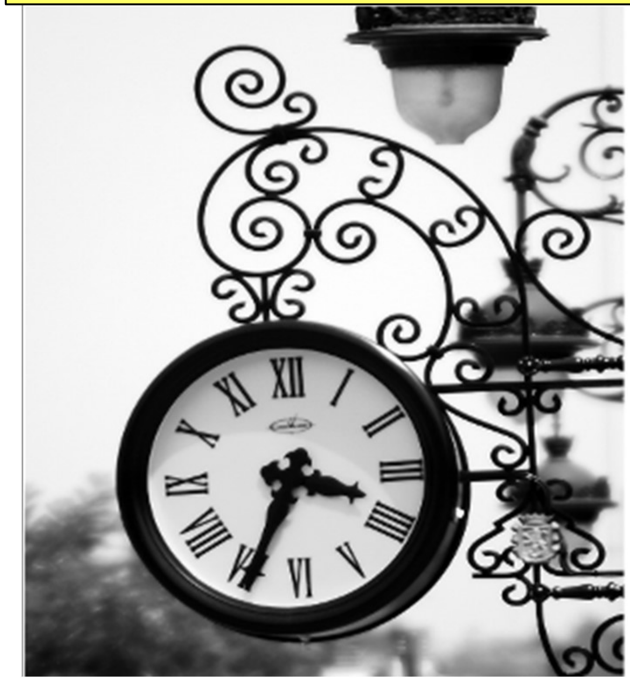

Results:

More **adherence to treatment**

Positive assessment of the physician

Consequences for the physician:

Closeness *to the patient*

Work satisfaction

THE CONSULTATION TIME DOES NOT INCREASE

Recuerde

- **Los antibióticos no son eficaces contra los resfriados ni la gripe**
- **Tome los antibióticos de forma responsable y sólo cuando se los recete el médico**
- **Todos somos responsables de conseguir que los antibióticos sigan siendo eficaces**

Cuando los *necesite*, utilice los antibióticos de manera responsable

Cuando le receten antibióticos, siga las instrucciones del médico para minimizar el riesgo de desarrollar bacterias resistentes.

Si no sigue las instrucciones correctamente —si, por ejemplo, acorta la duración del tratamiento o toma una dosis menor o no toma los antibióticos en los momentos correctos que le haya prescrito el médico—, las bacterias pueden adquirir resistencia a los antibióticos.

Las bacterias resistentes pueden quedarse en su organismo y también pueden transmitirse a otras personas. Esto le expone a usted y expone a otros al riesgo de no responder a los antibióticos la próxima vez que los vuelva a necesitar.

- **Siga las instrucciones de su médico y tome los antibióticos cuándo y cómo él se lo indique**
- **No utilice los antibióticos que le sobren**
- **Pregunte a su médico o farmacéutico cómo debe tirar los antibióticos que le sobren**

Si desea más información sobre el uso responsable de los antibióticos, visite: http://antibiotic.ecdc.europa.eu

EUROPEAN ANTIBIOTIC AWARENESS DAY

A European Health Initiative

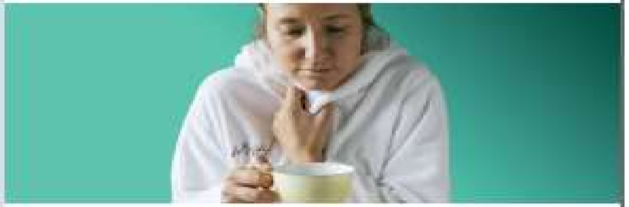

Uso responsable de los antibióticos

Contribuya a mantener la eficacia de los antibióticos

FONDO EUROPEO DE DESARROLLO REGIONAL
"Una manera de hacer Europa"

European Centre for Disease Prevention and Control (ECDC)
Tel: +46 (0)8 5860 1000
Email: EAAD@ecdc.europa.eu

Current Situation

Patient pressure	Communication skills<br>Deferred Prescription<br>Supporting material 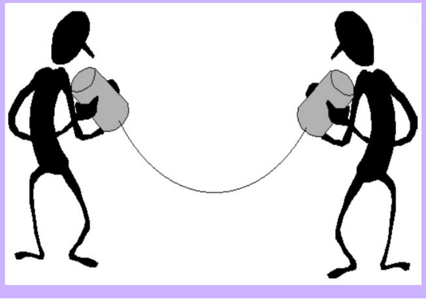
Fear / Uncertainty	Deferred Prescription 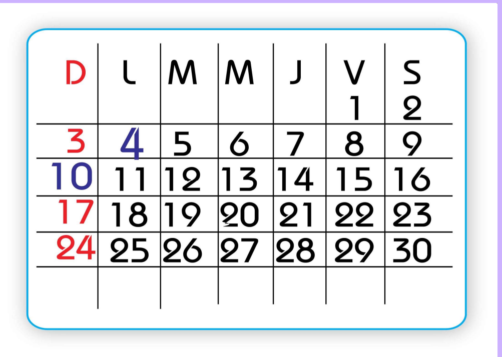
Adherence to clinical guidelines	

Delayed antibiotics for respiratory infections (Review)

Spurling GKP, Del Mar CB, Dooley L, Foxlee R

THE COCHRANE COLLABORATION®

This is a reprint of a Cochrane review, prepared and maintained by The Cochrane Collaboration and published in *The Cochrane Library* 2011, Issue 1

http://www.thecochranelibrary.com

WILEY
Publishers Since 1807

blinding gave two trials (Chao 2008; Little 2005a) which blinded the outcome assessor to give an odds ratio for these two trials of 1.42 (95% CI 0.92 to 2.19). The one completely unblinded trial (Little 1997) gave an odds ratio of 1.49 (95% CI 0.70 to 3.19). In the *delayed* antibiotic arm 413 of participants were satisfied or very satisfied out of 473 participants (87.3%) compared to 387 out of 465 participants in the *no* antibiotics group (83.2%).

DISCUSSION

Summary of main results

Small differences were found between prescribing strategies for clinical outcomes with *immediate* antibiotics most likely to show

Copyright © 2011 The Cochrane Collaboration. Published by John Wiley & Sons, Ltd.

benefit over *delayed* antibiotics in participants with sore throat and AOM. All strategies appear to have similar safety with no advantage found for *delayed* antibiotics over *no* antibiotics for disease complications. *Delay* and *no* antibiotic strategies dramatically reduce the use of antibiotics for ARTIs compared to *immediate* antibiotics. The least antibiotic use was in the *no* antibiotic group followed by *delay* and then *immediate*. The number needed to treat to prevent one antibiotic prescription using the delay strategy is 1.6 compared to *immediate* antibiotics. The number needed to treat to prevent one antibiotic prescription using a *no* antibiotic strategy compared to *delay* is 5.6. Patient satisfaction was highest in the *immediate* antibiotic group with 92.2% being satisfied or very satisfied with the consultation. The *delay* and *no* groups had similar quite high satisfaction rates at 87.3% and 83.2%, respectively.

Overall completeness and applicability of evidence

Dowell 2001; Little 2005a; Spiro 2006), the outcomes assessor was blinded but not the patient nor the care giver.
Otherwise, studies were well reported and appeared to be high quality.

Potential biases in the review process

Heterogeneity of RCTs is the main limitation of this review. Heterogeneity may have resulted from variable clinical presentations, differences in delay method, differences in antibiotic use and quality of included studies. Type I error is a concern when interpreting the results of this review, given the heterogeneity of results with multiple outcome measures. This is especially concerning for the comparisons for clinical outcomes between *delayed* and *immediate* antibiotic groups.

Agreements and disagreements with other

THE COCHRANE COLLABORATION®

Delayed antibiotics for respiratory infections (Review)

Spurling GKP, Del Mar CB, Dooley L, Foxlee R

	EFFECTIVENESS	SECURITY
DEFERRED Vs IMMEDIATE	≈	Increase in RAM M.N1<br>Increase in resistances

M.N1 a qué corresponde RAM?

MALLAH ... NARMEEN; 06/10/2020

Current Situation

Patient pressure	Communication skills<br>Deferred Prescription<br>Supporting material
Fear / Uncertainty	Deferred Prescription
Adherence to clinical guidelines	Online course

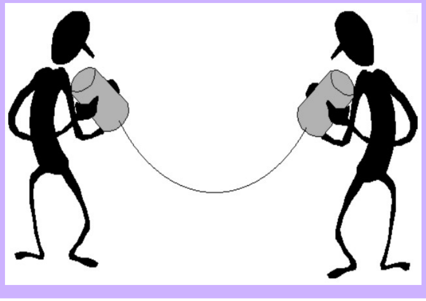

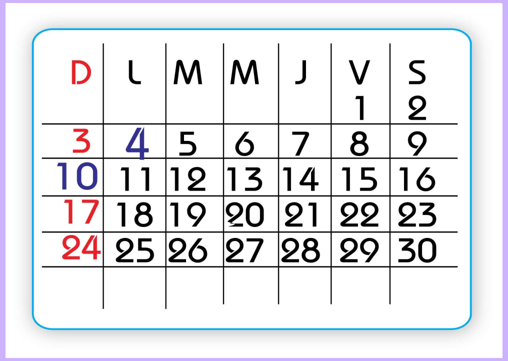

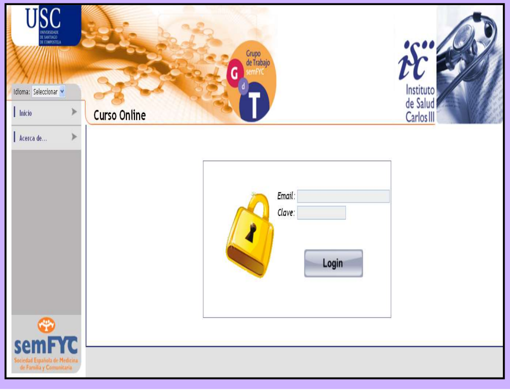

USC
UNIVERSIDADE DE SANTIAGO DE COMPOSTELA

Grupo de Trabajo semFYC

Instituto de Salud Carlos III

Idioma: Seleccionar

- Presentación
- Información General
- Metodología
  - Manual del usuario
  - Sistema de Evaluación
- Casos Clínicos
- Algoritmos
- Bibliografía de apoyo

semFYC
Sociedad Española de Medicina de Familia y Comunitaria

Sistema de Evaluación

Sistema de Evaluación

El curso está dividido en 5 casos clínicos que servirán de introducción a los conceptos teóricos. Se realizará una prueba de evaluación al final con preguntas tipo test, en la que el alumno tendra la oportunidad de valorar su evolución contrastando sus propias respuestas antes y después de realizar los casos.

Requerimientos exigidos para la acreditación

Haber superado en la prueba de evaluación final el 50 % de aciertos.

USC Universidade de Santiago de Compostela

Grupo de Trabajo semFYC

Instituto de Salud Carlos III

Idioma: Seleccionar

Inicio

Acerca de...

Curso Online

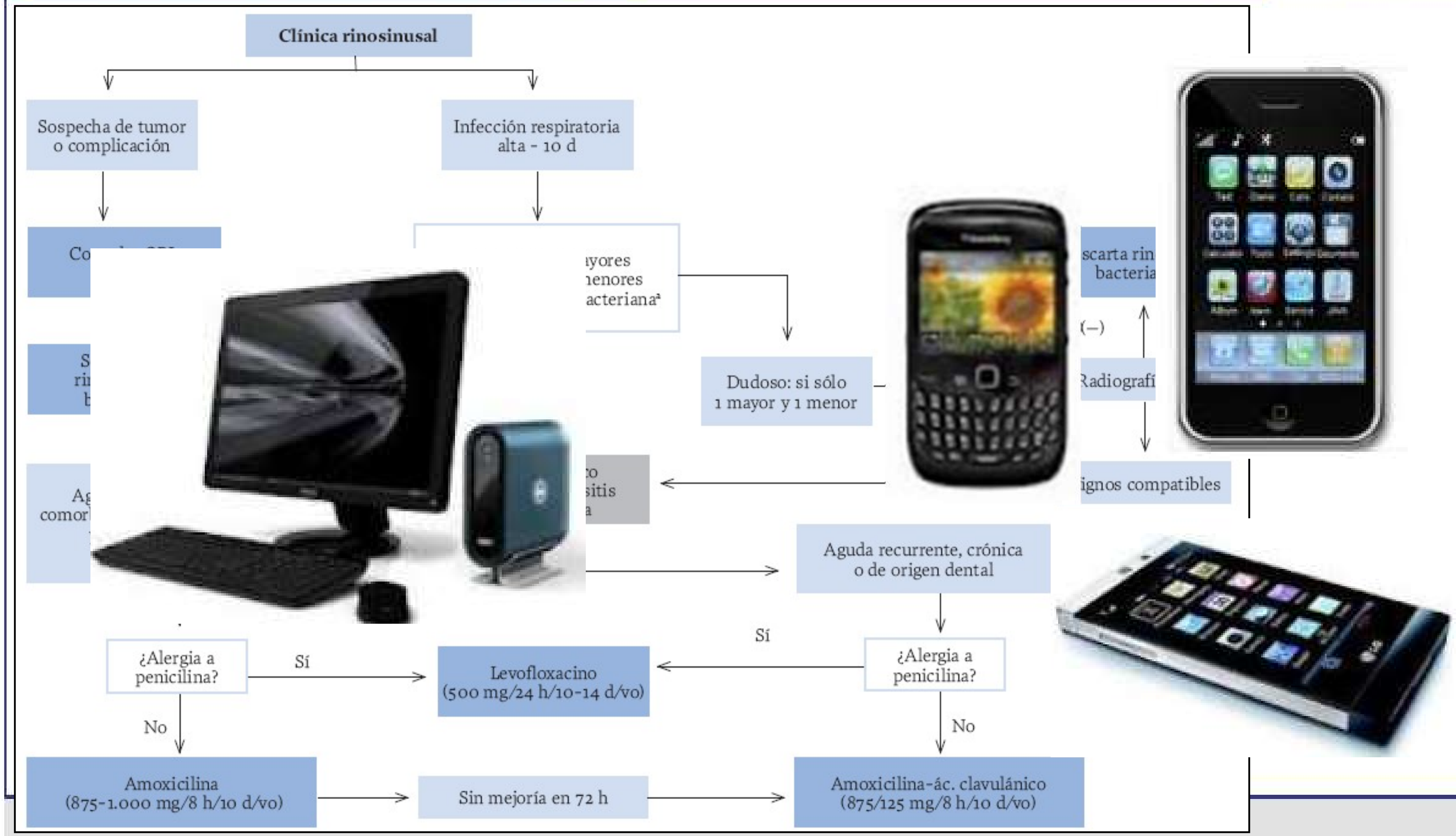

semFYC Sociedad Española de Medicina de Familia y Comunitaria

Sistema experto

Presión del paciente	Habilidades de comunicación<br>Prescripción Diferida<br>Materiales de apoyo
Miedo / Incertidumbre	Prescripción Diferida
Adherencia a guías clínicas	Curso online

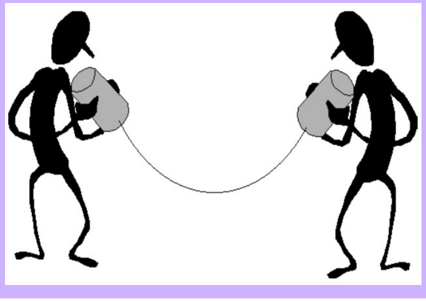

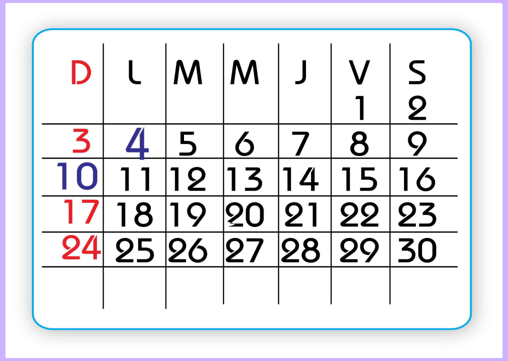

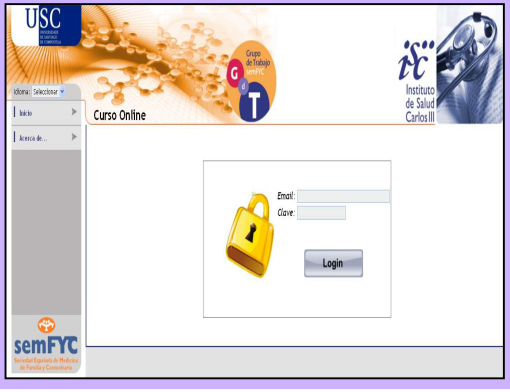

USC
UNIVERSIDADE
DE SANTIAGO
DE COMPOSTELA

MINISTERIO
DE CIENCIA
E INNOVACIÓN

Instituto
de Salud
Carlos III

FONDO EUROPEO DE DESARROLLO REGIONAL
"Una manera de hacer Europa"

MUCHAS GRACIAS POR SU ATENCIÓN

cristian.gonzalez@usc.es
